# Patients’ trajectory from tooth loss to dental rehabilitation and living with implant-supported dentures – a qualitative interview study

**DOI:** 10.1186/s12903-025-06072-5

**Published:** 2025-05-10

**Authors:** Gabriele Müller-Mundt, Philipp-Cornelius Pott, Lara Prinz, Simone Schaumann, Meike Stiesch, Nils Schneider, Franziska A. Herbst

**Affiliations:** 1 Institute for General Practice and Palliative Care, Hannover Medical School, Carl-Neuberg-Strasse 1, 30625 Hannover, Germany; 2 Clinic for Prosthetic Dentistry and Biomedical Materials Science, Hannover Medical School, Hannover, Germany

**Keywords:** Oral health, Dental implants, Health services research, Qualitative research, Patients

## Abstract

**Background:**

Since their introduction, implant-supported dentures have gained increasing popularity and are associated with high expectations. Nevertheless, as with other invasive procedures, implant treatment involves post-surgical risks as well as the risk of long-term complications. The present study aimed at exploring patients’ experiences of the entire trajectory of dental implant-treatment, to reach a profound understanding of the patients’ perception of the course of their journey from tooth loss to living with implant-supported dentures.

**Methods:**

The single-site study employed an exploratory, qualitative design with semi-structured interviews conducted with patients who had received implant-supported dentures for different indications at least one year prior. Thirty-three patients were consecutively recruited by dentists during regular check-ups from March to September 2023 at a German university dental clinic. Interview data were analyzed using deductive-inductive qualitative content analysis.

**Results:**

Of the 33 patients five patients did not return the consent-to-contact form, and one withdrew their consent, resulting in a final sample of 27 patients (82%). The participants held high expectations regarding the function, durability, and – in cases involving visible tooth gaps – aesthetic outcomes of their implants. Key concerns influencing treatment decisions included the need for bone augmentation and transplantation, age-related factors, and procedural risks. Notwithstanding the lengthy, stepwise nature of the treatment process, the considerable symptom burden, and the high financial cost, most patients considered the implant treatment worthwhile and felt well informed. With one exception the majority of participants (96.3%) reported that their implant-supported dentures functioned and appeared similar to their natural teeth. However, while satisfied with their implant-supported denture, two participants (7.4%) experienced persistent suffering due to temporomandibular disorder and persistent pain following treatment. The issue of prolonged facial pain has arguably been overlooked in aftercare of these patients.

**Conclusions:**

Dental implants are generally viewed as preferred option for oral rehabilitation. The results underline the importance of comprehensive counselling and after care irrespectively of the complexity of the treatment. While iatrogenic complications and the risk of their chronification are rare, careful attention to these risks remains essential. Further research, encompassing prospective longitudinal studies, is needed, given the risk of recall bias.

**Clinical trial registration:**

Does not apply.

**Supplementary Information:**

The online version contains supplementary material available at 10.1186/s12903-025-06072-5.

## Background

In high-income countries, the proportion of edentulous adults has substantially declined over recent decades [1–3]. Improved oral health care, alongside advancements in dental restoration and tooth replacement, has made visible dental gaps increasingly rare. Nevertheless, given the aging population, demand for dental restoration and replacement is expected to persist and possibly increase [4].

The majority of individuals who experience tooth loss have their teeth replaced with conventional removable partial or complete dentures. However, although these prostheses are frequently regarded as a “gift”, the adaptation to living with them is often characterized as challenging, and is associated with discomfort and distress (e.g [5]). In contrast, implant-supported dentures are perceived as more like natural teeth and are viewed favorably in terms of functionality, stability, and aesthetic appeal [6]. Subsequent to their introduction, implant-supported dentures have gained popularity and become increasingly accessible, at least for those patients who can afford the co-payment associated with dental implants in most countries. Dental implants are more costly than conventional dentures, and the stepwise implant treatment takes quite a long time. In addition, like any invasive procedure, implant treatment involves general post-surgical risks and complications, such as nerve damage, implant failure, and peri-implantitis [7, 8].

In Germany, epidemiological studies such as the German Oral Health Study (DMS) [9] and the Study of Health in Pommeria (SHIP) have shown that, over time, edentulousness has decreased while the prevalence of both healthy and restored teeth has increased [10]. Longitudinal findings from the SHIP have revealed a decline in missing and unrestored teeth across all age groups. For example, the use of double crown-retained partial removable dental prostheses increased from 15 to 20% between the 1997–2002 study period (SHIP-0) and the 2008–2012 follow-up (SHIP-2). Also the proportion of individuals with fixed implant-supported dentures rose significantly, from 0.16 to 2.5% over the same period [10]. The DMS indicated a comparable trend among individuals aged 65–74, with the prevalence of implant-supported dentures increasing from 0.7% in 1997 (DMS-III) to 8.1% in 2014 (DSM-V) [11]. According to the German Association of Implantology, approximately 1.3 million dental implants are placed annually in Germany [12]. The statutory health insurance scheme does not provide comprehensive coverage for the cost of dentures. However, depending on the specific tariff, privately insured patients, as well as those with statutory health insurance, may have conventional dentures and dental implants partially or fully covered through an additional private dental insurance. Nevertheless, given the higher out-of-pocket costs compared to conventional dentures, primarily socioeconomically well-off people opt for dental implants. DMS-data indicate that among younger seniors (age group: 65 to 74 years), those with a high and a medium social status were twice likely to have implant treatment for dental restoration than patients with a low social status [11]. Research on patients’ experiences of tooth loss and dental implant treatment has mainly relied on quantitative measures assessing functionality, aesthetics, and oral health-related quality of life [13]. However, a deeper understanding of patients’ perceptions of tooth loss, restorative treatment, aftercare, and living with implant-supported dentures is needed to optimize dental implant treatment. In recent decades, qualitative research on dental care has increased globally, including studies focused on implant treatment [14, 15].

Internationally, several qualitative studies have explored different aspects and/or stages of the dental trajectory from the patient perspective, including experiences of tooth loss [16, 17], motivations and expectations concerning implant therapy [18, 19], the implant treatment process itself [20], experiences of living with implant-supported prostheses [21], and experiences with complications such as peri-implantitis [22, 23]. Some studies have specifically explored the experiences of distinct patient groups, such as functionally impaired patients [24] and patients with a history of periodontal disease [25]. However, there remains a notable gap in the literature following the comprehensive trajectory of patients’ dental histories, from their initial tooth loss to their everyday life with implant-supported dentures [25, 26]. This lacuna is particularly evident in Germany, where, to the best of our knowledge, no studies have yet explored this full trajectory.

### Research aim and objectives

The present study is, to the best of our knowledge, the first qualitative exploration pertaining to the comprehensive trajectory of dental patients’ experiences of tooth loss and implant treatment in Germany. The study aimed at:

- identifying patients’ expectations, clinical and informational needs, and challenges in the course of the trajectory,
- describing and understanding patients’ utilization and perception of dental services related to dental implant therapy, with a focus on appropriateness at various stages of the trajectory.

The primary research questions posed were:

- *Expectations*: What were patients’ expectations of implant therapy and aftercare to maintain the success of the implant therapy?
- *Needs and challenges*: What did dental patients in need of dental rehabilitation after tooth loss perceive as their main clinical and informational needs as they progressed from the onset of tooth decay and tooth loss to dental rehabilitation and living with dental implants? What were the patients’ needs and expectations for dental aftercare in the event of long-term complications?
- *Use and perception of services*: What dental services were used by the patients at various points in their trajectory, and was this perceived as appropriate and adequately addressing their needs?

A more profound understanding of the patients’ perspectives and needs over the course of their journey can assist in advancing dental care and implant therapy to align more closely with patients’ needs.

## Methods

### Design

The study employed an exploratory, qualitative design [27], applying semi-structured interviews and a deductive-inductive approach to data analysis, to gain an in-depth understanding of patients’ experiences of and perspectives on the dental implant process and life with dental implants. The study followed the COREQ criteria for reporting qualitative research [28].

### Study context

The study was conducted as part of a collaborative project between the Clinic for Prosthetic Dentistry and Biomedical Materials Science (CfPD) and the Health Services Research Group of the Institute for General Practice and Palliative Care (IfGP) at Hannover Medical School, Germany. This paper draws on interviews with patients at the CfPD who had received implant-supported dentures and continued to attend the university dental clinic for follow-up care. The interview study was conducted in conjunction with an ongoing longitudinal observational study at the CfPD concerning the long-term success of dental implant treatment, which was supplemented by a patient survey assessing oral health-related quality of life [29].

### Ethical considerations

Ethical approval for the extension of the existing research project to include the interview study was obtained from the Ethics Committee of Hannover Medical School, Hannover, Germany (No. 8703_B0_K_2019). The purpose and scope of the interview study were thoroughly explained to each patient, and interested individuals received further written information. All participants provided written informed consent.

### Research team

The multi-disciplinary research team consisted of four dentists with expertise in clinical dental research (PCP, LP, SSch, MS); one physician specialized in family medicine with a broad expertise in public health and health services research (NSch); and two social scientists with extensive experience in health services research and qualitative methods, specialized in sociology and public health (GMM) and medical anthropology (FAH).

### Methodological approach

In order to achieve an in-depth understanding of the patients’ needs and perceptions, a qualitative study was deemed the most suitable approach. The study is based on semi-structured interviews, as this interview format has been proven to be the most suitable for the purpose of exploring individuals’ subjective experiences [30]. Due to the open-ended questions and the flexible handling of the interview guide, it offers the chance to explore issues that are pertinent to individual respondents but may not have been anticipated by the research team [30].

In the present study, the interview guide was developed based on a review of relevant literature and the combined expertise of the multi-professional research team. Key themes included patients’ dental health history, experiences with tooth loss, treatment decision-making, perceptions of the treatment and healing process, aftercare, and practical and emotional experiences of life with their implant-supported denture(s) (see Supplement Table S1 for the detailed interview guide). A supplementary socio-demographic questionnaire (16 items) was used to gather further information on participants’ age, gender, marital status, living situation, educational attainment, occupational status, and household income, as well as basic data on their reasons for receiving implant-supported denture(s), type of denture(s), and treatment history at the dental clinic.

To ensure the interview guide’s clarity, appropriateness, and capacity to generate rich data, it was piloted with three patients with implant-supported dentures who were not receiving treatment at the university dental clinic. Each of the three participants took part in an individual pilot interview conducted by one of two experienced researchers (FAH, GMM) who also conducted the interviews during the data collection period of the study. Two of the pilot interviews were conducted face-to-face and one was conducted by telephone. The participants were invited to provide verbal feedback regarding the comprehensibility and length of the interview, and any aspects they felt were missing. The pilot interviews were reviewed and discussed by the research team at the IfGP. There were no significant differences between the face-to-face interviews and the telephone interview. Following the critical review of the pilot interviews, the interview guide was adapted accordingly (e.g., simplification of the narrative impulse to start the interview, reformulation of questions to facilitate deeper exploration of critical topics). The data from the pilot interviews were discarded and not included in further data analysis.

### Participants and data collection

The study targeted adult patients aged *≥* 18 years who had received fixed or removable implant-supported dentures, regardless of the cause for tooth loss, and who continued to receive routine dental care at the CfPD at Hannover Medical School. Eligibility required that implant treatment had been completed at least 1 month prior to the data collection. Exclusion criteria included insufficient German language proficiency, cognitive impairment limiting the ability to provide informed consent, and/or manifest dental phobia. Patients who were actively participating in other CfPD studies were invited to participate.

As this was a qualitative study, a statistical sample size was not calculated prior to data collection. Sample size was determined during the research process on the basis of theoretical data saturation. The collection of data was concluded once data saturation was obtained. Data saturation was defined as being achieved when conducting further interviews no longer brought additional insights to the research question [31, 32].

Participant recruitment was conducted by CfPD dentists consecutively during regular patient appointments over a 6-month period, from mid-March to mid-September 2023, until relative data saturation was achieved. Patients were informed verbally by the dentists about the nature of the study and were invited to participate. Those who expressed initial interest were provided with detailed written information about the study, a consent form, the socio-demographic questionnaire, and an oral health-related quality of life survey (not analyzed in the current study). Both verbal and written communications emphasized the voluntary nature of the participation, patients’ right to withdraw without justification, and the confidentiality of their involvement. To ensure privacy, patients’ names and addresses were not disclosed to the members of the research team conducting the qualitative interviews. Interested patients returned a “consent to contact” form and/or directly contacted members of the IfGP research team.

Two researchers experienced in qualitative health services research and uninvolved in patients’ dental care (GMM, FAH) conducted the interviews. Patients could opt for either a telephone or a face-to-face interview at a location of their choice. Prior to the interview, participants were encouraged to ask any questions they might have. They were informed that the data collected from the interviews would be pseudonymized and they were further reassured that CfPD research team members would not have access to any identifiable interview data.

### Data management and analysis

All interviews were digitally recorded, transcribed verbatim, and pseudonymized by members of the IfGP health services research group. Two authors (GMM, FAH) cross-checked the transcripts for accuracy. Qualitative data management and coding were supported by the software package MAXQDA Version 18 (VERBI GmbH, Berlin, Germany), while descriptive statistical analysis of the socio-demographic data was conducted using IBM SPSS Statistics 26 (SPSS Inc, Chicago, IL, USA).

An explorative deductive-inductive approach was employed to qualitatively analyze the interview data [33]. Initially, two authors (GMM, FAH) independently reviewed and coded the transcripts using a code system developed in accordance with the main topics of the interview guide. During the initial coding phase, additional data-driven (sub-)codes emerged inductively and were subsequently discussed and refined through consensus-building. One researcher (GMM) then applied the finalized code system to the entire dataset. To ensure consistency, members of the research team conducted a comprehensive coding check, resolving any ambiguous assignments of text coding through team discussion until consensus was reached. In the total sample of 599 coded text units, 514 codings were found to be consistent, thus yielding an inter-coder agreement percentage of 85.8%. The remaining 85 codings (14.2%) exhibited minor discrepancies, predominantly arising from the allocation of text units to higher-level codes or specific sub-codes.

## Results

### Patient interviews and sample characteristics

Of the 54 patients who participated in the oral health-related quality of life survey, 33 (61%) provided written informed consent to participate in the qualitative interview study. However, five patients did not return the consent-to-contact form, and one withdrew their consent, resulting in a final sample of 27 patients (82% of the initial 33 patients).

The majority of participants opted for a telephone interview (*n* = 24, 88.9%). Those who expressed a preference for a face-to-face interview included one patient who was interviewed at home and two who were interviewed in an office at the IfGP. In six telephone interviews, the interviewee’s spouse was present but did not interfere. Some interviewees asked their spouse for reassurance, for example if they were unsure about when an event happened or who exactly was involved. Interview durations ranged from 12 to 32 min, with a median length of 17 min.

In relation to the interview format, it was observed that all three interviewees who expressed a preference for face-to-face interviews (11.1%) possessed an academic background and a sophisticated manner of expression. With an average duration of 25 min (range 18 to 32 min) the face-to-face interviews found to be marginally longer in comparison to the telephone interviews, which lasted on average 18 min (range 12 to 28 min). No significant differences in the response behavior of the interviewees were evident when comparing face-to-face interviews with participants who also had an academic background. Thus, the richness of the information gathered was comparable, apart from the fact that visual non-verbal cues like facial expression and body language are not conveyed in telephone interviews. For an overview of specific advantages and disadvantages of face-to-face and telephone interviews see e.g Oltmann [34].

Of the 27 interviewees, 13 were female and 14 were male, with a median age of 69 years (range: 49–91 years). Most participants were married and/or cohabiting with a partner (*n* = 20, 74%) and held academic qualifications (*n* = 20, 74%). Employment status varied, with 8 participants (30%) employed and 19 (70%) retired. Approximately three-quarters (*n* = 20) reported a relatively high monthly net income/pension exceeding 3,600 euros. Regarding health insurance, 11 participants were covered by statutory health insurance, while the majority (*n* = 17, 63%) were privately insured. Among those privately insured, 9 (71%) were (former) state servants eligible for state aid with healthcare costs in conjunction with a private health insurance. Additionally, 12 patients (44%) held complementary dental insurance to help cover co-payment costs for dental restoration and dentures (see Table 1 for detailed demographic and insurance information).

**Table 1:** Sample socio-demographic characteristics (*n* = 27)

Characteristic	Specification	
Gender	Female	13 (48.1%)
	Male	14 (51.9%)
Age	Median (*range*)	69 (49–91) years
Marital status	Single	1 (3.7%)
	Married/living with partner	20 (74.1%)
	Divorced/separated	4 (14.8%)
	Widowed	2 (7.4%)
Living with others or alone	Living with spouse/partner/children	21 (77.8%)
	Living alone	6 (22.2%)
Highest level of general education completed	Secondary general or intermediate school leaving certificate	5 (18.5%)
	Intermediate school-leaving certificate	22 (81.5%)
Professional training	Vocational training (dual system)	2 (7.4%)
	Full-time vocational school	2 (7.4%)
	Craftsman and technical school	3 (11.1%)
	University of applied sciences degree	3 (11.1%)
	University degree	17 (63.0%)
Occupational status	Employed	8 (29.6%)
	Retired	19 (70.4%)
Net household income (missing:* n* = 1)	*≤* 2,600 €	2 (7.7%)
	2,601 to 3,600 €	4 (15.4%)
	3,601 to 5,000 €	7 (26.9%)
	*≥* 5,001 €	13 (50.0%)

#### Participants’ dental history and treatment

Most participants had undergone their initial implant treatment several years prior (median: 7 years) and had previously received other types of dental restoration, including crowns, bridges, and/or conventional removable dentures. With the exception of one participant, all had received their most recent implant treatment at the university dental clinic. Major indications for the implant treatment were tooth loss due to decay and/or inflammation, and the need to replace previous dentures (e.g., broken crowns or bridges, suppurated crowned teeth). Two patients reported having lost single teeth due to periodontitis, and another two had a tooth broken by accident. Three patients underwent a reconstructive treatment after tumor surgery of the jaw. While the sample appears to be quite homogenous with regard to the patients’ socio-economic status (predominantly academic background and medium to high income), the participants represent a range of different indications for and types of implant-supported dentures, ranging from single dental implants to complete fixed or removable implant-supported dentures (see Table 2).

**Table 2:** Participants’ (*n* = 27) dental history and treatment

Characteristic	Specification	
Indication for implant-supported denture(s)	Tooth loss due to decayed and/or inflamed teeth	7 (25.9%)
	Wobbly teeth/tooth loss due to periodontitis	2 (7.4%)
	Tooth loss due to failed root treatment	1 (3.7%)
	Tooth broken due to (sports) accident	2 (7.4%)
	Replacement of previous denture needed (e.g., crown/bridge broken, crowned tooth suppurated)	7 (25.9%)
	Reconstruction after tumor-related surgical removal of part of the jaw (e.g., ameloblastoma)	3 (11.1%)
	Unspecified tooth loss/extraction	5 (18.5%)
Type of denture(s)	Implant-supported (single) crown(s)	14 (51.9%)
	(Single) implant-supported bridge	3 (11.1%)
	(Partial) implant-supported prosthesis	4 (14.8%)
	Implant-supported crown(s) and bridge/prosthesis	6 (22.2%)
Time since beginning treatment at the MHH dental clinic ^†^	Median	8 years
	Minimum/maximum	1 month to 42.5 years
Time since first needing an implant-supported denture	Median	7 years
	Minimum/maximum	10 months to 40 years
Time since first receiving an implant-supported denture	Median	4 years
	Minimum/maximum	1 month to 39 years
Time since last dental check-up (*missing*: *n* = 3)	Median	1 month
	Minimum/maximum	1 month to 6 months

† One participant attended the dental clinic following implant treatment in a dental practice

### Findings: patients’ perceptions of their trajectory from tooth loss to dental rehabilitation and living with implant-supported dentures

Patients’ experiences and perceptions of their treatment trajectory revolved around their: (1) dental history, experiences of tooth loss, and search for optimal restorative treatment; (2) decision to receive implant-supported dentures and perceptions of the implant treatment and healing process; and (3) experiences of living with implant-supported dentures, including care requirements and long-term complications. Table 2 summarizes these dimensions and the associated codes and sub-codes (see Table 3, with Supplementary Table S2 detailing the complete list of codes and sub-codes).

**Table 3:** Perceptions and experiences of the trajectory from tooth loss to living with implant-supported dentures (codes and sub-codes)

Dental history and search for optimal restorative treatment		Decision to receive implant-supported dentures and perceptions of the implant treatment		Living with implant-supported dentures	
Oral health and tooth loss – causal attribution	Treatment information and expectations	Treatment decision and concerns	Treatment procedure and healing process	Perceived outcomes	Care requirements and complications
■ Conceivable tooth loss – long history of dental problems ■ Immediate unexpected tooth loss	■ Dental counseling ■ Information behavior ■ Expectations of the dental implant treatment	■ Decision in favor of dental implant treatment ■ Concerns and fears ■ Cost considerations	■ Perceptions of the treatment procedures ■ Perceptions of symptoms and healing process	■ General outcome ■ Specific outcomes: Appearance and function	■ Handling and self-care ■ Professional dental care ■ Complications and aftercare

#### Theme 1: dental history and search for optimal restorative treatment

##### Subtheme: oral health and tooth loss – causal attribution (see Supplementary Table S3 for exemplary quotes)

Most patients reported a long history of dental issues and various restorative procedures preceding their tooth loss. Some patients referenced a *“hereditary predisposition”* (P22) as a contributing factor. Older patients, in particular, indicated cohort-specific influences, such as growing up during or shortly after the Second World War, when access to *“enough healthy food”* (P22) and adequate dental care *“comparable to today’s standards”* (P23) was limited. Some patients attributed their tooth loss to inadequate dental care, including oversights during routine check-ups (e.g., failure to monitor caries progression). Finally, some (predominantly male) patients acknowledged that their tooth decay and/or periodontal disease was the consequence of their *“youthful sins”* (P11), reflecting a previous disregard for oral health.

In five cases, tooth loss occurred unexpectedly. Two patients attributed their loss to a (sports) accident, while three others experienced tooth loss as a consequence of an oral tumor (*“My teeth suddenly became wobbly”* [P19]) and/or the effects of surgical tumor treatment: *“the entire lower jaw was removed*” (P06).

##### Subtheme: treatment information and expectations (see Supplementary Table S4 for exemplary quotes)

Most participants sought an implant consultation on the advice of their general dentist or relatives/acquaintances, particularly in cases where they were not initially treated at the dental clinic (as in the case of oral cancer patients).

The majority of patients reported that they had received comprehensive information and advice about treatment options, particularly regarding implant therapy. They noted that they were able to *“ask everything”* (P24) and appreciated being given *“enough time to think about it”* (P04) by their dentists. Some patients actively sought additional information: *“Of course I also did some research”* (P23), while others sought an independent second opinion to confirm *“whether [they should] actually let it be done”* (P23). Of note, male participants often relied on online resources for additional information: *“Well*,* I Googled around”* (P13), whereas female participants appeared more likely to consult their informal social networks: *“I just asked friends”* (P06). Several patients expressed a high level of trust in their dentist, relying on their advice *“without any questioning”* (P05). However, some patients who took a more passive approach (and especially those who later experienced treatment-related complications) reflected that they were perhaps too “naïve” and should have sought more independent information.

Patients’ expectations of the dental implant treatment (see Supplementary Table S5 for exemplary quotes). The decision to undergo implant treatment was frequently accompanied by high expectations regarding functionality, longevity, and, to a lesser degree, aesthetics.

In terms of functionality, expectations were especially pronounced among cancer patients who required dental reconstruction following tumor surgery. These patients expressed a desire to *“just eat and chew normally”* (P06) and regain the ability to *“bite strongly again”* (P12) with durable teeth. For many, the primary hope was that the implant-supported denture would mimic natural teeth in function and appearance. Some patients explicitly stated that they wanted to avoid flexible dental prostheses with a palatal plate in the upper jaw.

Aesthetic considerations varied in accordance with the location of the missing teeth. While patients missing molars in the posterior stated that *“appearance was actually less important”* (P21), those needing restorations in the visible anterior valued the opportunity to regain a *“natural”* and improved appearance.

Longevity was a significant expectation for most patients, who viewed implant therapy as a long-term solution. Older patients, in particular, expressed the hope that the implant-supported denture(s) would serve as a definitive solution to their dental problems, while younger patients highlighted implants as the *“easiest to maintain and best*” option. However, they acknowledged that there was *“no guarantee”* the implants would *“last forever”* (P26).

#### Theme 2: decision to receive implant-supported dentures and perceptions of the implant treatment

##### Subtheme: treatment decision and concerns

Decision in favor of dental implant treatment (see Supplementary Table S6 for exemplary quotes).

Patients differed in their levels of involvement in the treatment decision-making process. Some made the decision through a collaborative, shared decision-making process with their dentists, while others primarily trusted their dentists’ recommendations. Many patients expressed a strong aversion to conventional removable dentures, for both functional and aesthetic reasons, citing negative associations with their (grand-)parents’ experiences with such devices.

For patients who had undergone oral tumor surgery, an implant-supported prosthesis with bone grafting was seen as the only viable option: *“There was no other option than such a replacement”* (P06). Single-tooth replacement patients generally preferred implants over conventional tooth-retained bridges or removable partial dentures, to prevent damaging intact neighboring teeth: “*The decisive factor was that for a dental bridge*,* for example*,* you would have had to grind down healthy teeth”* (P16).

Beyond clinical recommendations (“*It was simply recommended to me as a better option”* [P22]), dental implants were commonly perceived as the *“simplest*,* safest*,* easiest to care for and best”* (P27) solution. Some patients also emphasized that they *“did not want a conventional prosthesis”* (P25), as they would struggle to adapt to flexible dentures. In particular, the palatal plate associated with conventional upper dentures was perceived as highly uncomfortable: “*The most unpleasant thing would be to have a plate on the palate”* (P09). For patients who first experienced temporary flexible dentures during the implant treatment process, this experience often reinforced their decision to opt for fixed implant-supported solutions.

Regardless of whether patients had lost one tooth or several teeth, they hopefully expected implant-supported dentures to function like their natural teeth *(“I simply had the expectation that it would really be almost like my own.”* P03).

Concerns and fears (see Supplementary Table S7 for exemplary quotes).

Most patients reported minimal anxiety about the treatment, describing it as *“normal surgical anxiety that something might get infected and something unpredictable might happen that normally shouldn’t happen*” (P21). Many felt reassured by being *“in good hands”* at the dental clinic. However, a few participants disclosed a persistent fear of medical procedures stemming from traumatic childhood experiences. Additionally, some patients were concerned about pre-existing health conditions that could elevate surgical risks and/or interfere with healing. For instance, patients taking medications such as bisphosphonates expressed worries about potential interactions that *“might interfere with the implant-supported dentures”* (P24). Bone augmentation with grafting was particularly concerning for some patients, who described it as the most frightening part of the procedure. Noteworthy, apart from pronounced concerns and fears expressed by participants in the context with a required bone augmentation and transplantation, there were no differences evident in the extent of treatment-related fears with regard to different types and the extent of the implant-supported dentures needed. While some participants did not harbor treatment-related fears, they were anxious about the possibility of the implant failing, leaving them with tooth gaps or conventional dentures – an outcome they hoped to avoid. In our sample, the fear *“it might not work”* was most pronounced among older patients with *“receding bones”* (P03).

Cost considerations (see Supplementary Table S8 for exemplary quotes).

In Germany, while basic dental care is covered by both statutory and private health insurance, dental restorations and dentures almost always require substantial co-payments. Patients generally acknowledged that dental implant therapy is *“of course expensive”* (P04), making the decision partly a *“question of cost”* (P07), since *“the health insurance company pays almost nothing*” (P07). Most participants were financially stable, and several had supplementary dental insurance, often acquired well in advance, in anticipation of future dental needs: *“Thank God I have a supplementary dental insurance. I got it very early because I knew I would get problems with my teeth at some point*” (P22). Even those without high incomes and/or supplementary dental insurance recognized that implant treatment, while expensive, was ultimately *“affordable”* (P16) and a valuable investment: *“Of course it’s a lot of money that you have to pay. […] But it was still worth it*” (P15).

##### Subtheme: treatment procedure and healing process

Perceptions of the treatment procedures (see Supplementary Table S9 for exemplary quotes).

The treatment process varied depending on the number of teeth replaced and whether preliminary procedures such as bone augmentation, bone grafting, or jaw reconstruction after oral tumor surgery were required. Overall, patients described the implant treatment as a lengthy, step-by-step process that *“took a while”* but, paradoxically, *“went very quickly”* (P15). Despite its duration, most participants emphasized that the treatment *“worked perfectly”* (P09) and *“was well coordinated”* (P25). Many patients appreciated that each step of the treatment was clearly explained to them, which helped them understand what to expect: *“When I was being treated*,* they told me what was going to happen next and so on”* (P01).

For patients who required more extensive procedures, such as bone grafting or reconstruction post-tumor surgery, the process was described as particularly challenging. While most stated that these procedures *“went smoothly”* (P23), some experienced them as “*very stressful”* (P14), describing the implant placement as a procedure they *”had to endure”* (P18). In line with this patients who received multiple dental implantats for fixed or removable prosthesis described the procedures more burdensome and difficult to manage. Although no patient of our sample reported pain during the procedure, some remarked that it was easy to *“underestimate”* the physical toll of multiple dental sessions, each requiring them to keep their mouth open wide “*so that the dentist can work in there as well as possible”* (P04). One patient described the surgical experience, and particularly the moment of sitting in the chair with their head covered by sterile cloths, as an *“overwhelming moment”* (P26) that felt oppressive.

Perceptions of symptoms and the healing process (see Supplementary Table S10 for exemplary quotes).

Most participants retrospectively described their post-operative symptoms, such as temporary swelling, pain, and facial bruising, as *“quite normal”* (P17) and generally less burdensome than anticipated. Moreover, many reported that everything *“healed without problems”* (P19). Although patients experienced some initial limitations, with some feeling *“handicapped at first”* (P25), they were generally well-informed (*“knew before”* [P27]) about the likely symptoms and found the experience manageable. As one patient noted, following “*the instructions exactly”* (e.g., applying cooling measures and eating only soft foods) made the symptoms bearable, and *“after a few days it was fine”* (P22).

For most, the healing period between treatment steps was temporarily limiting but not especially distressing. However, a subset of patients found the recovery phase long and challenging. This was particularly the case for patients who required a bone transplant for augmentation or an orthodontic surgical reconstruction following oral tumor surgery.

#### Theme 3: living with implant-supported dentures

##### Subtheme: perceived outcomes

General outcome (see Supplementary Table S11 for exemplary quotes).

Overall, the majority of patients were highly satisfied with both the process and the outcome of their implant treatment. They viewed dental implants as a well-considered choice, describing them as *“the simplest*,* safest*,* easiest to maintain and best”* (P27) solution for their dental needs. Patients who had previously experienced (temporary) conventional dentures particularly appreciated their *“well-anchored”* (P25) implant-supported dentures, which they felt positively contributed to their quality of life: *“It’s a completely different quality of life for me”* (P11). Even those who encountered a stressful treatment process and complications, such as persistent pain, emphasized that the implant *“itself is completely trouble-free”* (P10). Thus, irrespectively of the complexity of the dental reconstruction and number of lost teeth patients in our study pronounced being *“happy”* having *“well anchored”* dentures (P25).

While some patients perceived their implant-supported denture as their *“own tooth”* (P04), if not an *“integral part of the body”* (P23), others mentioned that they occasionally “noticed” the implant(s), such as when brushing their teeth. Only one participant described her implant-supported crown as a foreign body compared to her other (crowned) teeth: *“It just feels different from the other crowns”* (P05).

Most patients expressed that they *“would choose an implant again”* (P21) and *“can only recommend it to everyone”* (P03). However, a few noted reservations about repeating the treatment, particularly in consideration of age-related factors. One patient remarked that, from her perspective, implant therapy is *“a question of age”* and that, at an advanced age, one has to *“think about it again very carefully”* (P22).

Aesthetic outcome (see Supplementary Table S12 for exemplary quotes).

Many participants were pleased with the aesthetic results, describing their implant-supported dentures as looking *“great”* (P01) and often indistinguishable from their natural teeth. Some even felt that their appearance had improved, especially compared to previous crowned teeth of varying shapes. However, a few patients were disappointed by minor aesthetic imperfections, such as crowns that did not fully match the shape and/or color of their remaining teeth, or the visibility of the implant’s base screw over time. these issues were generally tolerated when involving molars, but visible imperfections – particularly in the front teeth – had a significant emotional impact. For example, one patient who experienced uneven gum height following an incisor replacement described it as *“traumatic*,*”* explaining that she has *“not smiled since then anymore”* (P10).

Noteworthy, among the patients in our sample who were to some extend disappointed with the treatment outcome in terms of appearance were solely patients who received single implant supported crowns, while patients with more complex implant-treatment were mostly highly satisfied.

Functional outcome (see Supplementary Table S13 for exemplary quotes).

Functionally, most patients reported that their implant-supported dentures performed nearly *“perfectly”* (P12). Especially patients with fixed or removable partial dentures and prostheses emphasized being *“happy”* to be able to eat everything *“properly”* again (*“I can chew and eat now almost everything.”* P14). Many found their implants functioned like *“real teeth*,*”* allowing them to eat a full range of foods, including hard items such as crusty bread and apples. For patients who had experienced tooth loss as a catastrophic event, the ability to resume normal eating was deemed particularly valuable. However, some remained slightly cautious, feeling *“a little scared”* that the implant might fail: *“That’s because you’re branded when your teeth just broke off when eating just normally”* (P25). Some patients experienced minor functional adjustments, such as initially biting their cheek while chewing with the new implant-supported denture. One patient temporarily avoided hard foods due to severe jaw joint pain attributed to the extensive duration of the implant therapy sessions.

##### Subtheme: care requirements and complications

Handling and self-care (see Supplementary Table S14 for exemplary quotes).

The majority of patients recognized the importance of a rigorous daily oral hygiene routine, alongside regular professional cleanings and dental check-ups, as essential for maintaining implant-supported dentures. Patient perceptions varied regarding the additional effort required for implant maintenance compared to natural teeth, largely contingent on their prior oral hygiene habits. Some patients reported minimal adjustment, describing “*the handling just normal”* (P26) and continuing the same thorough routines they had previously practiced, including the use of interdental brushes and floss. For these individuals, implant care was simply an extension of their established hygiene practices. However, most patients acknowledged that their implants necessitated a more specialized approach to oral hygiene: *“Implants need to be cared for*,* of course”* (P11). A few patients stated that they received comprehensive instructions on implant maintenance (*“Of course we learned a lot that we didn’t know before”* [P23]) and that they strictly adhered to these recommendations: *“So I do everything that is discussed with me in the dental clinic”* (P22).

The perceived effort involved in maintaining implants varied. While some felt that the additional care requirements were minimal (*“That’s not a lot of effort”* [P25]), others described the routine as complex and time-consuming (“*It’s a real act*,* it takes a long time”* [P18]). Regardless of the effort required, most patients deemed the maintenance worthwhile, emphasizing a commitment to *“do whatever”* they *“can to keep the teeth in order”* (P01).

Professional dental care (see Supplementary Table S15 for exemplary quotes).

All patients were receiving ongoing care at the dental clinic, with most emphasizing the importance of regular professional cleanings and check-ups. Many reported that they had already been accustomed to routine dental maintenance before receiving implants, while others noted that their implant treatment had introduced a more structured approach to preventive care.

Frequency of appointments varied according to individual dental conditions. Most patients reported two or more dental clinic visits per year, though some attended only annually. Patients with a predisposition to or existing periodontal disease were under closer surveillance to prevent or control peri-implantitis, typically attending three to four times a year for check-ups and preventive treatments. Two patients indicated that their frequent dental visits placed them *“constantly under control”* (P18), ensuring the early detection and management of any issues. Conversely, two patients admitted to having recently neglected their dental care routine, recognizing that they *“should have gone there a little more often”* (P09).

##### Subtheme: complications and after care (see Supplementary Table S16 for exemplary quotes)

The majority of patients treated at the dental clinic reported no significant complications following their implant procedures. Some patients even highlighted the absence of long-term treatment-related issues, attributing their smooth recovery to a healthy lifestyle: *“Well*,* I actually live a healthy life and I don’t know if it has anything to do with that”* (P12). However, some patients did encounter complications post-treatment.

A few patients who had not exhibited any signs of periodontitis prior to the implant procedure subsequently developed peri-implantitis, characterized by gingival recession or inflammation around the implant site. These patients were placed under close dental monitoring and scheduled for frequent professional cleanings each year to manage the condition. One patient attributed their gum inflammation to a lack of diligence in oral hygiene, citing a failure to replace their toothbrush regularly: *“This [gum inflammation] had nothing to do with the implant. That was the toothbrush”* (P07). Two patients experienced implant loss due to peri-implantitis. These individuals expressed uncertainty about undergoing further implant treatment to replace the lost teeth, as they were molars and the tooth gap was not experienced as a significant issue. One older patient decided against further treatment *“Due to my age and no lack of functionality”* (P13), while another older patient was still contemplating the options: *“It was suggested that another implant could be placed*,* but I see it like this: once it fell out*,* once it loosened*,* hmm*,* I’m thinking about that at the moment”* (P04). Other complications included issues with implant-supported prostheses. One patient reported that the implant-supported removable prosthesis *“became slightly loose”* and this was subsequently *“corrected properly”* (P14).

In two cases, implant-supported single crowns *“broke off”* (P27). For one patient, this occurred shortly *“after the warranty period”* (P02), leaving uncertainty as to whether the broken screw could be replaced. For the other patient, the crown broke just three months after *“the whole [treatment] was finished*,*”* which was experienced as *“a little annoying*,*”* as *“everything [had] to be made again”* (P27).

Two patients experienced persistent jaw joint problems, including severe pain while chewing, jaw cracking, and difficulty opening their mouths, which they attributed to the prolonged therapy sessions that required extended mouth opening. Both successfully managed their symptoms with manual therapy initiated by their dentists at the university dental treatment center. Another patient, who had suffered massive postoperative symptoms such as pain, swelling, and bruising following a jaw bone transplant, suffered from persistent facial pain. This patient felt unsupported by the dental clinic, as no physical cause was identified, nor was a referral made to a specialist for chronic pain management, leaving the patient feeling *“pretty much left alone with it”* (P10).

## Discussion

### Summary of the results

For most participants, tooth loss stemmed from a long history of tooth decay and/or periodontal disease, which they attributed primarily to genetic predisposition and, to a lesser extent, inadequate oral hygiene and dental care during their youth. Many patients viewed dental implants as a favorable solution, based on their dentists’ recommendations and/or their active search for information across a variety of sources. Expectations for implant treatment were notably high, particularly concerning functionality, longevity, and – in cases of visible tooth gaps – aesthetics. The decision to pursue implant treatment generally involved a careful comparison of the advantages and disadvantages of dental implants versus conventional dentures, with patients often drawing on personal experiences or those of significant others. Beyond general anxieties associated with surgical interventions, concerns predominantly centered on bone augmentation and transplantation. The majority of patients indicated that they felt adequately informed about the details of the treatment process. Despite the extended, stepwise nature of the procedure, the associated symptom burden, and the substantial cost, the vast majority expressed that the benefits of implant treatment outweighed these drawbacks. Most participants reported that their implant-supported dentures both functioned and appeared similar to their natural teeth. While dental implants were broadly regarded as worthwhile, patients also weighed factors such as age and procedural risks in their decision-making. Patient adherence to oral hygiene and dental care seemed exemplary, even in cases involving complications. However, persistent pain with no identifiable physiological cause tended to be neglected in dental care. No measures were implemented to prevent the chronification of (sub-)acute postoperative pain or to address jaw joint problems. Specialist pain therapy consultations were not initiated, leaving patients with no effective multimodal pain management plan. Consequently, one patient experiencing persistent post-traumatic pain reported feeling “left alone.”

### Discussion of the results in relation to previous research

#### Tooth loss and causal attribution

In their review of observational studies, Gerritsen and colleagues highlighted a substantial body of evidence linking tooth loss to diminished oral health-related quality of life, impacting psychological and social well-being, especially based on the location of the missing tooth gap [35]. Previous qualitative research on tooth loss has similarly underlined the disruptive psychological and social effects of tooth loss, which patients often describe as highly detrimental and disruptive [17, 25, 26]. However, participants in the present study focused less on the psychological and social ramifications of tooth loss and instead emphasized the negative connotations they associated with conventional removable dentures as symbols of advanced age and physical impairment. Patients’ causal attribution of their tooth loss aligns with trends observed in high-income countries, where preventive care and tooth restoration have become central to dental health practices. In Germany, as in other high-income nations, there has been a notable shift towards a greater emphasis on preventive practices and efforts to restore teeth to an optimal state [2, 4, 9].

#### Expectations and information

The high expectations of implant patients regarding function and aesthetics align with the findings of previous qualitative and quantitative research [6, 36, 37]. Although studies have indicated that patients often hold unrealistic expectations and lack knowledge about ongoing maintenance needs [36, 38, 39], participants in the present study largely reported feeling well informed. In line with this findings of Kashbour and colleagues in their qualitative studies on treatment perception of patient who received different types of dentures and on information provision in dental implant treatment in indicated that the patients felt well prepared for the dental treatment procedures but in contrast to the accounts of the patients in the current study the patients studied by Kashbour and colleagues appeared to be less well prepared in terms of information and knowledge for the post-surgical healing and the maintenance phase [40, 41]. In their survey with patients at two clinical dental treatment centers Simensen and colleagues found that many patients lacked information on post treatment requirements [41]. These authors demonstrated a comprehensive understanding of essential oral hygiene practices and the regular care necessary for successful implant maintenance, including diligent daily oral hygiene, routine dental check-ups, and periodic professional cleanings [40–42]. Patients in the current study seemed to have received mostly quite comprehensive individually targeted information concerning the implant treatment and maintenance in the course of their dental care at the university dental clinic, stating with the offered special implant counseling. However, partly they lacked relevant information about rare risks on long-term iatrogenic complications such as chronification of post-surgicial facial pain (see below).

In line with the findings of Simensen and colleagues [41] and in contrast to the findings of Atieh and colleagues, where affordability emerged as a primary determinant in participants’ choice of restorative options due to the high cost of implant treatment [36], financial concerns were relatively minor for most participants in the current study, who were largely financially secure and/or had a special private additional dental denture insurance covering at least parts of the costs of implant-supported dentures which are not covered by the sickness fund in Germany.

#### Treatment and healing process

The findings of Osman and colleagues in their interview study with edentulous patients who receiving maxillary and mandibular implant-supported overdentures suggest that patients may experience greater physical trauma from the surgical placement of dental implants than they initially anticipated [43]. In contrast to the previously mentioned study [43] and in line with Kashbour and colleagues’ findings on patients’ experiences with implant surgery, who also interviewed patients with different extend of tooth loss [40], the majority of participants in the present study retrospectively reported feeling adequately informed and prepared for the surgical aspects of their implant treatment and most participants reported having tended to overestimated the physical discomfort associated with implant surgery. While Kashbour and colleagues stated that the patients underestimated the extend of post-surgical impairments [40], most patients in our study seemed to have been prepared adequately about the likely symptom burden they had to endure in healing phase and perceived them as manageable and less burdensome than anticipated. Similary, Nogueira and colleagues found that patients with single-implant mandibular overdentures considered the post-surgical healing period less burdensome than anticipated [44].

#### Treatment outcome

In accordance with the findings of several qualitative and quantitative studies, most participants in the present study expressed high satisfaction with the results of their implant treatment, citing enhanced quality of life following the procedure (e.g [22, 25, 45–47]). However, comparative quantitative studies have indicated that improvements in oral health-related quality of life with implants may not be significantly greater than those achieved through conventional dentures [46]. Moreover, qualitative research has shown that implant treatment is irrespectively of the complexity and extent frequently perceived by patients as a “normalization” process, whereby implants come to be regarded “just as normal tooth/teeth” and integrated seamlessly into the body (e.g [14, 18, 24]. This sentiment of normalization was echoed in the present study. Finally, in line with Atieh et al. [36], in their interview study on patients’ experiences with immediate single molar implants, the present study found no significant differences in perceptions of implant outcomes (in terms of function and aesthetics) between male and female participants or across different age groups.

#### Future decision and complications

The majority of participants in the present sample expressed a preference for dental implants and indicated that they would recommend the treatment to others. This adjustment was also evident in the study by Nogueira and colleagues among patients with single-implant mandibular overdentures [44]. However, while quite satisfied with their implant-supported denture(s) some older patients expressed reservations, citing the increased risks associated with advanced age. Consistent with findings from an Austrian survey, older adults (aged 50 and above) expressed greater skepticism towards implant treatment compared to younger age groups [39]. In a study by Ellis and colleagues, older patients dissatisfied with conventional dentures but hesitant about implant treatment described that heightened fear and anxiety, particularly regarding surgical pain and procedural risks, were their primary reasons for declining implants [48]. Additionally, like in our study some questioned the appropriateness of undergoing such a procedure at an advanced age.

While rare, implant treatment does carry the risk of iatrogenic complications that can significantly impair quality of life [45, 49]. A scoping review examining temporomandibular disorder (TMD) in the context of dental implant treatment yielded two key results: (1) prolonged therapy sessions during implant procedures may contribute to the development of temporomandibular joint issues, and (2) implant placement can alleviate TMD symptoms, particularly in cases of missing posterior teeth [50].

In the present study, some participants experienced TMD symptoms and persistent post-surgical pain, which caused substantial distress. These results emphasize the importance of recognizing both general surgical risks and specific factors that may contribute to the development of TMD, such as pre-existing arthrosis and the potential for post-surgical pain to become chronic. Rigorous post-operative monitoring is essential to address severe complications, including excessive bleeding, swelling, acute post-surgical pain, and any history of chronic pain or psychological conditions such as depression and anxiety, as these can adversely impact quality of life. Of note, even the participant who suffered considerable post-surgical pain expressed satisfaction with her dental implant’s function and appearance. However, she did suggest that, in hindsight, a discussion of potential long-term iatrogenic complications would have been beneficial. Devine and colleagues similarly emphasized that the risk *“of lifelong chronic post-surgical pain is rarely recognized or discussed during the consent process for dental procedures”* [45]. In line with these findings, early referral to a pain specialist is recommended for patients experiencing severe or prolonged pain following implant treatment.

### Limitations of the study

The primary limitation of the present study was its small sample size, drawn exclusively from a single university dental treatment center, which may limit the generalizability of the results to the wider population of implant patients. Consequently, further research with a more diverse patient cohort, including patients from a variety of socio-economic backgrounds, is essential to better inform service provision and improve patient-centered care. Nevertheless, with respect to the socioeconomic background, the composition of the present sample largely corresponds to the characteristics of dental implant patients in population-based dental studies, such as the DMS [9]. In addition, (self-)selection bias have to be considered. Patients with less favorable treatment outcomes may not have continued their dental treatment at the university dental clinic or may have been less likely to participate in the interview study. Another limitation arises from the retrospective nature of the data collection, as participants described their experiences across the entire dental implant trajectory, from their initial tooth loss to their treatment decisions and experiences receiving and living with implant-supported dentures. Thus, recall bias might have affected their descriptions and interpretations of events. A systematic review found that patients tended to retrospectively rate their pre-treatment quality of life lower than they did prior to implant treatment [13].

## Conclusions

When based on an informed decision and followed by a profound after care, dental implant treatment appears to be the preferred option for oral rehabilitation following tooth loss. Even though the patient’s journey from tooth loss to dental rehabilitation seems quite long and demanding, the effort involved in implant treatment is, in retrospect, considered worthwhile by most patients. The present study demonstrated that implant-supported dentures are subjectively experienced as comparable to natural teeth in terms of functionality and longevity, thus contributing significantly to improved quality of life. However, while rare, the potential for iatrogenic complications and the risk of their chronicity must be considered and communicated during the treatment planning process and after care.

When considering the risk of recall bias into account, the results underscore the necessity for prospective, longitudinal studies monitoring patients throughout the entire treatment process, commencing from the initial tooth loss.

## Electronic supplementary material

Below is the link to the electronic supplementary material.

Supplementary Material 1

## Data Availability

The raw data related to this publication cannot be openly released, as some restrictions due data protection regulations apply. The data contain transcripts of interviews and no interviewees consented to having the complete transcription of their interview shared. Extensive quotations from the interviews are included in the supplementary material to assist the reader in understanding the study findings and conclusions.

## Abbreviations

DGI: Deutsche Gesellschaft für Implantologie im Zahn-, Mundt und Kieferbereich e.V. (German Association of Implantology)
DMS: Deutsche Mundgesundheitsstudie (German oral health study)
CfPD: Clinic for prosthetic dentistry and biomedical materials science
COREQ: Consolidated criteria for reporting qualitative research
IfGP: Institute for general practice and palliative care
SHIP: Study of health in pommeria
TMD: Temporomandibular disorder
