# Supplementary material for: Patients’ trajectory from tooth loss to dental rehabilitation and living with implant-supported dentures – a qualitative interview study
Source: BMC Oral Health. 2025 May 10;25:705. doi: 10.1186/s12903-025-06072-5 (PMC12066058; doi:10.1186/s12903-025-06072-5)
Supplement: Supplementary file 1 — Supplementary Material 1 [file 12903_2025_6072_MOESM1_ESM.docx]

**Supplementary Material**

**Content**

Supplementary Table S1: Interview guide

Supplementary Table S2: Perceptions and experiences of the trajectory from tooth loss to living with implant-supported dentures (codes and sub-codes)

**Exemplary quotes – Theme 1: Dental history and search for optimal restorative treatment**

*Subtheme: Oral health and tooth loss – causal attribution*

Supplementary Table S3: Oral health and tooth loss – causal attribution

*Subtheme: Treatment information and expectations*

Supplementary Table S4: Treatment information – dental counseling and information behavior

Supplementary Table S5: Expectations of the dental implant treatment

**Exemplary quotes – Theme 2: Decision to receive implant-supported dentures and perceptions of the implant treatment**

*Subtheme: Treatment decision and concerns*

Supplementary Table S6: Decision in favor of dental implant treatment

Supplementary Table S7: Concerns and fears

Supplementary Table S8: Cost considerations

*Subtheme: Treatment procedure and healing process*

Supplementary Table S9: Perceptions of the treatment procedures

Supplementary Table S10: Perceptions of symptoms and healing process

**Exemplary quotes – Theme 3: Living with implant-supported dentures**

*Subtheme: Perceived outcomes*

Supplementary Table S11: General outcomes of the dental implant treatment

Supplementary Table S12: Outcomes in terms of appearance

Supplementary Table S13: Outcomes in terms of function

Supplementary Table S14: Handling and self-care

*Subtheme: Care requirements and complications*

Supplementary Table S15: Professional dental care

Supplementary Table S16: Complications and after care

**Supplementary Table S1:** Interview guide

| **(I) (Dental health) History, treatment decision, implant treatment, and healing process** |
| --- |
| 1. How did you come to have an implant? |
| *Optional in-depth inquiries:*   - How did you lose your tooth/need (a) denture/s? - Why did you choose an implant? - What information did you receive prior? From whom did you get this information? - What considerations and alternatives to dentures did you consider? What were the arguments for and/or against different types of dentures? - How did the implant treatment go? How did you experience the treatment and the healing process? (e.g., Did you experience any limitations due to the extent and duration of pain, swelling of the surgical wound, bleeding, or inflammation?) |
| **(II) Expectations (function and aesthetics)** |
| 2. What did you expect from the implant? To what extent have these expectations been met? |
| *Optional in-depth inquiries:*   - What were your hopes for the dental implant? - How important was it to you that the dentures would be anchored in the jaw? - What was particularly important to you about the appearance of your dentures? (Do you like the appearance of the denture/s? To what extent?) |
| **(III) Quality of life and handling** |
| 3. What is life like with the implant? |
| 4. How do you find the handling of your implant (e.g., daily care)? |
| *Optional in-depth inquiries:*   - To what extent have your expectations of eating with the implant been met? - Have your eating habits changed since receiving the implant? - To what extent have your expectations regarding dental care with the implant been met? - To what extent did you expect it to be like this or different? What has changed for you? |
| **(IV) Aftercare** |
| 5. How does the dental aftercare work for you?  *Optional in-depth inquiries:*   - How have your dental check-ups changed since receiving your dental implant? - To what extent did you expect it to be like this or different? |
| **(V) Worries and fears (complications)** |
| 6. What worried you?  *Optional in-depth inquiries:*   - Looking back: Did you have any fears before the treatment? To what extent did these fears materialize or not? - To what extent were you afraid of complications? Did complications occur? If so, how were they dealt with? |
| **(VI) Review and outlook** |
| 7. All in all: What was good? What was less good?  8. What would you have wished for?  *Optional in-depth inquiries:*   - Looking back: To what extent have your hopes for the implant been fulfilled? - Looking back: Why would you decide in favor of/against an implant? - How much do cost issues (e.g., reimbursement) affect your retrospective assessment? - What would the perfect dental implant look like for you? |
| **(VII) Closing** |
| 9. Is there anything about your implant I haven’t asked you? |
| 10. Is there any question I should have asked to better understand your experience? |
| 11. Do you have any questions? |
| ***Optional cross-topic, narrative-generating questions*** |
| - *Can you tell me about a situation that makes this clear?* - *Can you give me an example/tell me about a situation?* - *What was your most significant/positive/negative experience?* |

**Supplementary Table S2:** Perceptions and experiences of the trajectory from tooth loss to living with implant-supported dentures (codes and sub-codes)

| **Dental history, tooth loss, and search for information about dental rehabilitation options** | | **Decision to receive implant-supported dentures, process of implant treatment, and healing** | | **Living with implant-supported dentures** | |
| --- | --- | --- | --- | --- | --- |
| **Dental history – tooth loss and causal attribution** | **Information and expecta­tions about dental implants** | **Treatment decision and (remaining) concerns/fears** | **Experiences of treatment and healing process** | **General perceptions of dental implant treatment outcomes** | **Oral hygiene, dental care, and complications and aftercare** |
| **Immediate unexpected tooth loss**   - Accident - Oral tumor and surgical tumor treatment   **Conceivable tooth loss – long history of dental problems**   - Disposition and insufficient dental care - Insufficient daily oral hygiene and dental care - Long history of tooth decay - Treatment error | **Information and dental counseling**   - Comprehensive information and counseling - Insufficient counseling   **Information behavior**  *Active*   - Self-initiated dental implant counseling - Internet search and second opinion - Advice from relatives and friends   *Passive*   - Reliance on dentist advice   **Expectations of the dental implant treatment**  *Function*   - Function like own teeth - Able to bite powerfully again - Firm denture, no disturbing palatal plate   *Appearance*   - Preserved natural appearance - Improved appearance   *Longevity*   - Long-lasting, definitive solution - No guarantee to last forever | **Decision in favor of dental implant treatment**  *Pro implant*   - Best/only option - Equivalent to natural teeth - Neighboring teeth remain intact   *Contra conventional dentures*   - Disturbing – could not cope with it - Disturbing memories of (grand)parents’ conventional dentures - Disturbing experiences with flexible dentures   **Remaining concerns and fears**  *No concerns/fears*  *General fears*   - Hope nothing will happen - Traumatic experiences - General fear but trust that one is in good hands   *Specific individual fears*   - Individual disease or medication-related risks but trust that one is in good hands - Fear concerning bone augmentation with transplant - Fear it might not work   **Cost considerations**   - Affordability - Costly but worth the investment | **Perceptions of the dental implant treatment**   - Less demanding than anticipated – went great - Long, demanding process but well performed and coordinated - Stressful procedure that had to be endured   **Symptom burden and healing process**   - Minor normal temporary symptoms - Manageable symptoms and restrictions - Burdensome and difficult-to-manage symptoms | **General outcome**  *Implant best solution – regaining quality of life*   - Generally very satisfied - Having firm teeth again   *Integration into the body image*   - Integral part of the body - Foreign body   *Decision for or against an implant in the future*   - Would choose it again - Preferable option but carries considerable risks   **Appearance**  *Appearance great*   - Delightful appearance - Like natural teeth   *Appearance improved*   - Visual improvement - Teeth/crowns adjusted - Upper/lower jaw adjusted - Like new teeth   *Appearance imperfect if not* *impaired*   - Not perfect but hardly visible - Appearance impaired   **Function**   - Like natural teeth - Able to eat properly again - Normally but careful eating - Temporary restrictions | **Handling and self-care**   - Good oral hygiene as usual - Oral hygiene as recommended - Special oral hygiene needed but no effort - Oral hygiene worthwhile yet requires significant effort   **Professional dental care**   - Regular prophylaxis and dental control as usual - Close dental control and prophylaxis to prevent or treat periodontal disease - Constant dental treatment required - Special implant-related dental control and guidance - Neglected dental control and prophylaxis   **Complications and dental aftercare**  *No significant complications*  *Implant/denture failure or loss*   - Denture/crown does not fit - Crown broke off - Implant loss - Denture damaged during dental intervention   *Treatment-related persistent symptoms and impairment*   - Post-treatment jaw joint problems and therapy - Persistent pain – left alone |

**Exemplary quotes – Theme 1: Dental history and search for optimal restorative treatment**

*Subtheme: Oral health and tooth loss – causal attribution*

**Supplementary Table S3:** Oral health and tooth loss – causal attribution

| **(Sub-)Code** | **Exemplary quotes** |
| --- | --- |
| **Immediate unexpected tooth loss** | |
| Accident | I had a sports accident. I've been playing basketball for 40 years and sometimes things get a little rough. So that day I got an elbow in the face, on my front teeth. I noticed immediately that a tooth was somehow broken, loose, and went to the dental clinic relatively quickly because I had there for years already […]. It was then discovered that the tooth was really broken and that the tooth root had to be removed. (P17:3; male, age 70–74 years, implant-supported single crown) |
| Oral tumor and surgical tumor treatment | It was a tumor, epithelial carcinoma on the lower jaw. [...] Therefore, the lower jaw was removed and then rebuilt. (P06:2f; male, age 50–54 years, implant-retained fixed prosthesis)  My teeth suddenly became wobbly and I also noticed that I had bad breath, somehow, and also pain, well, but the dentist couldn't find anything. And he referred me to the A. [dental clinic]. And there it was immediately established that there was cancer behind it, yes. […] The entire left upper jaw was removed and teeth that were still present in the right upper jaw became wobbly and no longer stable during subsequent operations. They were then pulled out so that the entire upper jaw was now without teeth. Only in the lower jaw a few teeth were left. (P19:3, 50; female, age 90–94 years, implant-retained fixed prosthesis)  It was simply that I had an ameloblastoma [in the jaw], and it was then completely removed. (P03:3; female, age 55–59 years, implant-retained fixed partial dentures) |
| **Conceivable tooth loss – long history of dental problems** | |
| Disposition and insufficient dental care | My dentist at the time didn't actually provide good basic care, and probably I don’t have good teeth due to my family background, despite intensive oral hygiene. (P26:3; male, age 55–59 years, implant-supported single crowns)  I have had problems with my teeth since I was 13. I'm born in ‘48 and obviously my mother wasn't able to eat enough healthy food. And due to heredity, I had periodontal disease already at an early age. My father has had that, too. My mother no longer had any teeth when she was 40. […] Another problem was that I had the first teeth pulled while I was studying and my parents had not enough money to pay for any kind of denture. […] When we went to A. [town in southern Germany] in 1975 and earned money, I got my first denture. […] I think there is also a predisposition – I don't know whether my mother lost her teeth so early during pregnancies during the war. She had already dentures when she was 40. It wasn't that bad with my father, but he also had periodontal disease. I have siblings and a brother who also suffers severely from periodontal disease. (P22: 3–5, 82; female, age 75–79, implant-supported single crowns and implant retained fixed partial dentures) |
| Insufficient daily oral hygiene and dental care | It’s because I have mild periodontal disease due to a rather careless tooth brushing I had done for years, my teeth were a bit rotten then. Well, I attribute this to myself too. It’s my own fault. (P12: 29; male, age 50–54 years, implant-supported single crowns and implant-retained fixed partial dentures)  To be honest, It started rather early because of my youthful sins, the tooth roots were damaged and so my teeth had to be extracted little by little. (P11:46; male, age 65–69 years, implant-retained fixed prosthesis)  I somehow belong to a generation where parents didn't pay attention to dental hygiene that much. And that's why I rarely brushed my teeth, it's all somewhat rotten [...] tooth decay, all sorts of things and tartar and so on. And the gums have continued to recede. Some teeth then wobbled and somehow fell out. (P02:6; male, age 70–74 years, implant-supported single crowns) |
| Long history of tooth decay | The teeth simply were no longer in order, wobbly and carious, and were crowned already twice, [...]. That [the crowned teeth] wobbled and it didn't work anymore at all. I felt very uncomfortable. (P24:14, 30; female, age 80–84 years, implant-supported single crowns) |
| Treatment error | Well, I lost a molar in my opinion because the dentist didn't notice in time that it had a crack. That's why the tooth simply rotted away at some point. (P27:3; female, age 65–69 years, implant-supported single crowns)  A root canal treatment went wrong, the tooth didn't survive. (P16:3; male, age 45–49 years, implant-supported single crown) |

*Subtheme: Treatment information and expectations*

**Supplementary Table S4:** Treatment information – dental counseling and information behavior

| **(Sub-)Code** | **Exemplary quotes** |
| --- | --- |
| **Dental counseling** | |
| Comprehensive information and counseling | The care and information were comprehensive and completely sufficient for me, because we talked about it and I understood everything well. […] The risks that my bone is very thin, that I might need bone augmentation were discussed with me very well in detail. (P26:4–9; male, age 55–59 years, implant-supported single crowns)  Everything was fine, I could always ask anything and everyone encouraged me. (P24:55; female, age 80–84 years, implant-supported single crowns)  Everything has been addressed, [...] explained very clearly and not only the advantages but also the disadvantages [...]. I felt really well informed. (P21:9; male, age 75–79 years, implant-supported single crowns and implant-retained fixed partial dentures)  It was very important to me that I really had someone who could explain things to me, give me advice and enough time to think about it and also shows me the differences between alternative options. […] I was very well advised and [told] that it is a bit expensive but high quality, […] you could take something cheaper but it would be important to pay attention to quality. These were convincing arguments. And I found that very positive, too, I have to say. (P04:35–39; female, age 55–59 years, implant-supported single crown) |
| Insufficient counseling | I wasn't actually told what other options there were. I was simply told: “No, it has to be done like that.” […] So during the dental treatment I was told, a bone graft needs to be done using my own bone, foreign material is not possible and so on. Yes, I had the feeling that I had not been informed about what other options there were, besides bone transplant, […] what risks it actually entails. And, I didn't had any problems with the implant itself afterwards. But I had a lot of trouble with this bone transplant afterwards, it was terrible. (P10:11; female, age 55–59 years, implant-supported single crown)  I was there for a regular check-up and got along very, very well with the dentist who looked after me there. […] He just told me, which was correct, that there was nothing more he could do for me, that probably implants would have to be done. And the intention was very positive to arrange a consultation right now. That is very nice, but it's not always the right way for the patient. [I would have] simply needed a few days [to] think about how it could be treated if something happened, … I was simply overwhelmed. (P09:43–47; female, age 80–84 years, implant-retained fixed partial dentures) |
| **Information behavior** | |
| *Active*  Self-initiated dental implant counseling | Because of my petres adgredia disease, I had infusions and asked myself to what extent it was even possible for the implants to hold. So I went to the implant consultation. (P24:18; female, age 80–84 years, implant-supported single crowns)  I went to the implant consultation where everything was explained to me in detail. (P15:15; female, age 65–69 years, implant-supported single crowns and implant-retained fixed partial dentures)  My dentist wanted to do it [implant treatment] himself, but then I said: “Yes, maybe, let's see first.” […] I went to the A. [dental clinic] for consultation. (P02:10; male, age 70–74 years, implant-supported single crowns) |
| Internet search and second opinion | Well, you look on the internet, but actually I have talked it through with Dr. A. [dentist at the implant consultation of the dental clinic] relatively well. […] We talked over and over again about what we could do. (P11:7; male, age 65–69 years, implant-retained fixed prosthesis)  Well, I Googled around [to get informed], I knew what was coming up, and [there were] the medical consultations because there was basically no alternative, except a flexible denture and I didn’t wanted that at all. (P13:11; male, age 80–84 years, implant-supported single crowns and implant-retained fixed partial denture)  Well, I’m a natural scientist and I used all information available. At that time it wasn’t quite as great as it is today with the internet. […] And I then got a second opinion as to whether I should actually let it be done. (P23:11; male, age 70–74 years, implant-supported single crowns) |
| Advice from relatives and friends | I went to the implant consultation of the A. [dental clinic]. Actually, I came to the A. [dental clinic] through my husband. He had had an implant placed by Doctor A. [dentist at the dental clinic] and was enthusiastic about it. (P15:13; female, age 65–69 years, implant-supported single crowns and implant-retained fixed partial dentures)  When I heard that I needed bone augmentation, I first told my friends, who had implants. (P01:7; female, age 85–89 years, implant-supported single crowns)  I just asked friends if they could recommend a dentist who was good at placing implants and then, I have to admit, I walked in blindly. (P05:5; female, age 55–59 years, implant-supported single crown) |
| *Passive*  Reliance on dentist advice | I was a bit naïve the first time, believed my dentist that everything would be fine. (P27:35; female, age 65–69 years, implant-supported single crowns)  What other options and risks there are besides autologous bone transplantation were not discussed. But that was no issue at all. They said it had to be done this way. And you know, as a patient you are in a position where you somehow trust that everything is right they say. And so did I. That was very naïve. (P10:63; female, 55–59 years, implant-supported single crown)  It was just clear to me that I was going to get an implant and then that was okay. I didn't question anything further or got any additional information. (P05:5; female, age 55–59 years, implant-supported single crown) |

**Supplementary Table S5:** Expectations of the dental implant treatment

| **(Sub-)Code** | **Exemplary quotes** |  |
| --- | --- | --- |
| **Function** | |  |
| Function like own teeth | [I hoped] that it's just the same thing when chewing and eating as with a normal tooth. (P26:24; male, age 55–59 years, implant-supported single crowns)  I had the hope that I would be able to eat and chew normally again, almost as I did before. (P06:21; male, age 50–54 years, implant-retained fixed prosthesis)  The appearance was actually less important [...] this is not as conspicuous as the front area. Yes, the expectation was that I would have a functional chewing surface for a longer period of time, [...] this taking out and taking in is not my thing. (P21:29–33; male, age 75–79 years, implant-supported single crowns and implant-retained fixed partial dentures)  Well, I simply had the expectation that it would really be almost like my own. (P03:11; female, age 55–59 years, implant-retained fixed partial dentures)  The expectation (was) that this would solve the problem and put in a functional replacement, and that it would be done with it. (P13:27; male, age 80–84 years, implant-supported single crowns and implant-retained fixed partial denture) |  |
| Able to bite powerfully again | I only had the expectation that after two years I would finally be able to eat a loaf of bread again, to bite into a sandwich. (P19:17; female, age 90–94 years, implant-retained fixed prosthesis)  I didn't have any great expectations. [...] My expectation was actually that I would be able to bite powerfully again. (P12:41–45; male, age 50–54 years, implant-supported single crowns and implant-retained fixed partial dentures) |  |
| Firm denture, no disturbing palatal plate | If these implants had not been possible, the alternative would have been that I would get a normal prosthesis with a plate under the palate and so on […]. No, I didn't want a complete prosthesis. It should be a bit firmer. (P25:15; female, age 65–69 years, implant-retained removable prosthesis)  […] and then this plate, that was actually the most unpleasant thing to have a plate under the palate. […] The problem was the palatal plate. (P09:11; female, age 80–84 years, implant-retained fixed partial dentures) |  |
| **Appearance** | |  |
| Preserved natural appearance | Yes, that [appearance] was also important to me. […] With increasing age at some point the varnish is off, but that what you can be saved a little without doing anything new, so I would never have anything done surgically. Not that. But the fact that it looks like my own teeth, that was somehow important to me. (P09:13; female, age 80–84 years, implant-retained fixed partial dentures)  I had also read about it on the internet before and it was then said, that practically the implant is then like a real tooth. (P08:14; male, age 65–69 years, implant-supported single crowns)  Well, in principle, adapted to the shape of the other teeth. […] that it really fits to the rest of the teeth. (P05:25;58; female, age 55–59 years, implant-supported single crown) |  |
| Improved appearance | Of course, I also hoped for a visual improvement. (P26:25; male, age 55–59 years, implant-supported single crowns)  […] he pulled my teeth a little bit up, ... Now I have a really good facial image. Top and bottom match, it (the mouth area) didn't sink in, as you often have when you get a dental prosthesis. So it's become a completely normal facial image. (P25:19; female, age 65–69 years, implant-retained removable prosthesis) |  |
| **Longevity** | |  |
| Long-lasting, definitive solution | Of course, [I hoped] that it lasts a long time and doesn’t become loose, as is often the case with hip or knee operations or something like that, where the implants become loose and so on. (P18:39; male, age 80–84 years, implant-supported single crowns)  The expectation [was] that the [dental] problem would be solved and a functional denture would be inserted, and that it would be done with this. (P13:26–29; male, age 80–84 years, implant-supported single crowns and implant-retained fixed partial denture)  I was hoping that it would last a really long time, I'm 74 and I won't live that long anymore so that I can finally draw a line under this whole dental thing, that it somehow lasts permanently. (P02:16; male, age 70–74 years, implant-supported single crowns) | |
| No guarantee to last forever | In my opinion, it [the implant] is the simplest, safest, easiest to maintain and best. […] Of course there are no guarantees. […] The probability is high that it may not last forever. (P26:37; male, age age 55–59 years, implant-supported single crowns) |  |

**Exemplary quotes – Theme 2: Decision to receive implant-supported dentures and perceptions of the implant treatment**

*Subtheme: Treatment decision and concerns*

**Supplementary Table S6**: Decision in favor of dental implant treatment

| **(Sub-)Code** | **Exemplary quotes** |
| --- | --- |
| **Pro implant** | |
| Best option | It [implant treatment] was simply recommended to me as the better option. (P22:12; female, age 75–79, implant-supported single crowns and implant-retained fixed partial dentures)  My jawbone is receding, so a bridge would be suboptimal, they said, so a better alternative would probably be implants. (P26:3; male, age 55–59 years, implant-supported single crowns)  Because there was no tooth behind it anymore, I was told that a bridge wouldn't work at all and whether I could imagine doing that with an implant. [… ] The tooth had also been fitted with a crown and so I decided to do that and said, “Well, I'll just get an implant, that's better than not having a tooth, you also need a certain amount of chewing surface.” (P04:6–7; female, age 55–59 years, implant-supported single crown) |
| Only option | That was because of the epithelial carcinoma and that was basically the decision, that's how it [reconstruction of the jaw and dentition] will be done. [… ] There was no other option than a replacement like this. (P06:13, 38; male, 50–54 years, implant-retained fixed prosthesis)  There was no alternative because the hole in the mouth was too big […] it was the only alternative for me. (P19:35–37; female, age 90–94 years, implant-retained fixed prosthesis)  At some point he [dentist] said: “Well, that’s no longer possible! In order to get stability in there, we have to do a crown build-up or something else, a bridge build-up, that doesn't hold up anymore. … We have to put implants in.” (P23:3; male, age 70–74 years, implant-supported single crowns) |
| Equivalent to natural teeth | For me, it (the implant) is like my own tooth. (P04:15–17; female, age 55–59 years, implant-supported single crown)  I wanted to have real teeth equivalent to my own. (P18:103; male, age 80–84 years, implant-supported single crowns) |
| Neighboring teeth remain intact | We thought about it for quite a long time. […] if you had made a bridge, then you would have had to grind down healthy teeth, for example, or something like that. (P16:5; male, age 45–49 years, implant-supported single crown)  Honestly, I have to say, I think it was really great for the reason what Mr. A [dentist] also said at the time: “The other teeth on the right and left are not affected.” (P03:15; female, age 55–59 years, implant-retained fixed partial dentures) |
| **Contra conventional dentures** | |
| Disturbing – could not cope with it | That [the fixed denture] is important to me because taking it out and in is not my thing. […] It [the implant] is more like a part of the body, I mean, the implants were never a foreign body for me. But everything else is a foreign body and something different emotionally. (P21:30–33; male, age 75–79 years, implant-supported single crowns and implant-retained fixed partial dentures)  The implant is the better solution for me, because with removal dentures, that would be even more of a hassle for me. [...] It's expensive but I think it's affordable for me. (P16:22–25; male, age 45–49 years, implant-supported single crown)  Flexible dentures would mean a significant restriction on my life, [...] if not the worst case, [...] if needed I would have another implant inserted, even with bone augmentation. (P13:43–47; male, age 80–84 years, implant-supported single crowns and implant-retained fixed partial denture)  Actually the most unpleasant thing would be having a plate on the palate. (P09:10; female, age 80–84 years, implant-retained fixed partial dentures)  I'm not that old, at least at that time I didn't feel that old. If this implant hadn't been possible, the alternative would have been to get a normal removable prosthesis with a plate on the palate and so on. [...] No, I didn't want a conventional prosthesis. It should be a little bit firmer. (P25:14–15; female, age 65–69 years implant-retained removable prosthesis) |
| Disturbing memories of (grand)parents’ conventional dentures | And I always wanted to have a solid set of teeth not something to take out, because I always saw my grandma and grandpa when the evening came and they would take them out and then they would be in the water glass. It was so terrible for me to see that as a young person. So I said, you never want to do that. That's why it was always clear to me: only solid implants. (P18:7; male, age 80–84 years, implant-supported single crowns)  I had no experience at all because I didn't have dentures yet. But that's one of those childhood memories, when I think of my grandparents, that really disgusted me as a little child, that these teeth, how should you say, this dental structure had to be taken out, it was somehow attached with brackets to the own teeth. […], then there was a palatal plate and I found it all very unpleasant, and I couldn't imagine having something like that in my own body. [...] That was basically the reason why I went on this implant track. [...] Adhesive powder: drugstores and pharmacies are also full of these aids that have to be used in order to somehow be able to keep this artifact in the mouth. (P17:42–48; male, age 70–74 years, implant-supported single crown)  I still remember my mother. She had beautiful teeth. But then they had to be pulled, little by little. Then she always had this fuss with this replacement and said always: “I can't eat anything, it all tastes like algae or metal.” Then they had to be renewed, she was in pain, and really experienced something with it. That’s why I thought myself: “Never!” [...] Dr. B. [dentist at the dental clinic] gave me a temporary prosthesis with brackets. [...] I couldn't cope with it at all. I will never, never will I have a denture like that. [...] Messing around with it, would be terrible for me! Since I experienced that with my mother. That won’t work for me at all. (P01:14–17; female, age 85–89 years, implant-supported single crowns) |
| Disturbing experiences with flexible dentures | Well, I (tried) a conventional denture first, although Dr. A. [dentist at the dental clinic] said: “I would do an implant.” But I said, for cost reasons and so on, it costs 9,000 euros, I said: “Then I’ll try a conventional denture.” That was nothing, […] wasted time. (P07:4–8; male, age 70–74 years, implant-supported single crowns)  I had a temporary one-tooth prosthesis made because I thought to myself, “Okay, you want to bite properly and not bite into a hole.” But I couldn't get along with this prosthesis at all. […] Actually it wasn't a relief, rather it made biting more difficult. Putting it in and out was unpleasant for me. That was the reason why I said, “Okay, I’ll try an implant.” (P05:3–5; female, age 55–59 years, implant-supported single crown)  Well, I also had a temporary denture, which was attached right and left to molars. […] Well, first: it doesn’t always sit properly. Then I also had the problem with this temporary solution that I was very sensitive to the weather. I always noticed that right away. Then, of course, food residue got stuck underneath. So these are things that I can't say in any way about the implant. (P03:5; female, age 55–59 years, implant-retained fixed partial dentures)  I couldn't cope with these temporary dentures at all. Well, a final prosthesis probably is different, but when I was alone here at home, I took the thing out, […]. It was always a foreign body. […] I didn’t get used to it. […] I can come to terms with things that aren't particularly nice *laughs* but I couldn't come to terms with that. (P09:3–7; female, age 80–84 years, implant-retained fixed partial dentures) |

**Supplementary Table S7:** Concerns and fears

| **(Sub-)Code** | **Exemplary quotes** |
| --- | --- |
| **No concerns/fears** | I’m completely relaxed about it. (P12:47; male, age 50–54 years, implant-supported single crowns and implant-retained fixed partial dentures)  No, I didn‘t have that [fears]. (P06:35; male, age 50–54, implant-retained fixed prosthesis)  No, no, I didn't have that [any fears]. Honestly, I have to say, I really felt like being in good hands there (in the dental clinic) right from the beginning. (P03:33; female, age 55–59 years, implant-retained fixed partial dentures)  I got used to it. Manipulations in the mouth aren't particularly pleasant, not that great but I have come to terms with it to some extent. I don't break out in a sweat when I have an appointment at the dental clinic. It's okay. (P09:28–29; female, age 80–84 years, implant-retained fixed partial dentures) |
| **General fears** |  |
| Hope nothing will happen | Of course, I hoped that there would be no problems at the surgical site, such as pain in the jaw, in the eye socket, and so on, that nothing like that would occur so far. (P26:25; male, , age 55–59 years, implant-supported single crowns)  This general fear that one has about whether things will go well. (P18:11–15; male, age 80–84 years, implant-supported single crowns) |
| Traumatic experiences | I'm always afraid of operations, hospitals and so on. That's because I was in the hospital for a long time as a child, that's a horror for me. And I'm afraid of pain, too, I have to admit. (P15:56–57; female, age 65–69 years, implant-supported single crowns and implant-retained fixed partial dentures)  Well I was in the hospital as a little boy for a whole year because I had a very complicated arm fracture, [...] whenever I notice certain smells, I get a crisis. (P02:48; male, age 70–74 years, implant-supported single crowns) |
| General fear but trust that one is in good hands | First, I was a bit naïve. I believed my dentist that everything would go well. After I heard a lecture from Mr. A. [university professor at the dental clinic] and knew what could go wrong, I thought to it's better to put yourself in the hands of experts. With Mr. A. [university professor at the dental clinic] I had zero concerns or doubts that it would not go well. (P27:32; female, age 65–69 years, implant-supported single crowns)  Well, I generally have fears about surgery. That was actually to be done in the operating room there in jaw surgery. [...] I'm always afraid of surgical procedures because the problem of germs is always virulent, not that it might not work. Of course, it can always be the case. You're tense that it hopefully works. But these weren't huge fears because I actually felt being in good hands [...] as I said, the normal surgical fears that something could become infected and something unpredictable might occur that normally shouldn't happen. [...] It was like a technique for me and if I have the feeling that the people who use the technique know what they're doing, then that's no big problem for me. (P21:64–71; male, age 75–79 years, implant-supported single crowns and implant retained fixed partial dentures) |
| **Specific fears** | |
| Individual disease or medication-related risks but trust that one is in good hands | Then I cringed because it was a real operation. [...] Our dentist was currently expanding his practice at an implant center. But at the time I had tremendous problems with my circulation and my heart was racing. And then I said to him: “I want to go to a facility so that I don't have to sit there in the treatment chair with you if something happens to me during the operation or something like that.” [...] And so I went to A. [dental clinic] for implant counseling and that convinced me. I then practically went to the dental clinic for treatment with the implants. [...] I was incredibly reassured when I was pushed into a real operating room, with my heart problems at the time. If something would happened to me, I could be saved in a neighboring department of the clinic. In addition, my surgeon, who came from the same city, was also an emergency doctor. [...] Well, all conditions were so great, I felt completely safe and everything went smoothly. (P23:3–9; male, age 70–74 years, implant-supported single crowns)  I had got an infusion-therapy because of my bone disease. And I had to find out to what extent it might interfere with the implant-supported dentures. [...] After I heard that the bisphosphonates dissolve the bones, I was worried that the implants wouldn't last. [...]. But as the professors (at the dental clinic) recommended it (an implant) and said it is possible, I have put myself trustingly in their hands. (P24:44; female, age 80–84 years, implant-supported single crowns) |
| Fear concerning bone augmentation with transplant | It was also said that if it [the implant] might not hold for some reason, then bone will be removed, [...] from the thigh, or elsewhere. [...] I was a little worried about that. (P24:44; female, age 80–84 years, implant-supported single crowns)  I think there were few alternatives. Well, I was worried about whether the bone would heal properly. And I would have found it really stupid if jawbone had to be transplanted from further back, or something like that. But that I was really scared or something like that, no, I wouldn't say that. (P16:18–19; male, age 45–49 years, implant-supported single crown)  I have to say, I was really scared, because a bone reconstruction was necessary. (P15:9; female, age 65–69 years, implant-supported single crowns and implant-retained fixed partial dentures)  So when I heard that the bone had to be build-up, I told it my friend. [...] They said: “Don't do that, you'll think the dentist will come out of your head.” [...] All I remember is that I was very afraid, terribly afraid because of the bone reconstruction. (P01:31–35; female, age 85–89 years, implant-supported single crowns) |
| Fear it might not work | Of course, I was afraid [...] that the jawbone might be already too thin, that it would be not possible to anchor it properly, or an infection might occur and the entire implant would have to be removed again. Well, I was a bit worried about that. (P04:24–27; female, age 55–59 years, implant-supported single crown)  Well, I know that my bone is receding and I'm just wondering how long this implant will last. When will this tooth [implant] fall out? (P05:47; female, age 55–59 years, implant-supported single crown)  No fears, I was just worried that it might not work, that it [the implant] won't heal in and that I will have to end my life that way [with missing teeth or removable dentures]. (P19:30–31; female, age 90–94 years, implant-retained fixed prosthesis) |

**Supplementary Table S8:** Cost considerations

| **(Sub)Code** | **Exemplary quotes** |
| --- | --- |
| **Affordability** | Of course, dental implant is a question of cost. […] Either you can afford it or you can't. It's just like that, you can’t help it. The health insurance pays almost nothing. (P07:55–61; male, age 70–74 years, implant-supported single crowns, GKV)  It just has to fit in financially […]. My children are older now, they no longer live at home. When they were 15, 16, 17, and it started with my jaw treatment and the costs, they kept saying, "No, Dad, it's all good." My wife is very accommodating anyway and says: "That's fine, we'll pay for it somehow!" […] But the financial aspect, the money, was indeed something to consider. (P26:47; male, age 55–59 years, implant-supported single crowns, Aid/pKV + suppl. insurance)  Thank God I have a supplementary dental insurance. I got it out very early because I knew I would get problems with my teeth at some point. […] We are doing quite well financially. […]. But so it went through the private supplementary dental insurance. (P22:73–80; female, age 75–79, implant-supported single crowns and implant-retained fixed partial dentures Aid/pKV + suppl. insurance)  I am entitled to state aid and have a supplementary private insurance. Therefore, I didn’t have to pay much extra. (P21:52; male, age 75–79 years, implant-supported single crowns and implant-retained fixed partial dentures, Aid/pKV + suppl. insurance)  The health insurance paid a lot and I had fortunately already taken out supplementary insurance beforehand. Thus, the costs were in the green area, so that we were able to shoulder them. (P19:38–39; female, age 90–94 years, implant-retained fixed prosthesis, Aid/pKV + suppl. insurance)  I'm glad that we have this additional insurance so that I didn't have to pay for everything myself... that was a good thing. They took over 40 percent or so. […] In terms of costs it was still possible, the health insurance company also paid something. (P15:65–69; female, age 65–69 years, implant-supported single crowns and implant-retained fixed partial dentures, GKV + suppl. insurance)  I have a relatively good private insurance. It covers 80 percent of the costs and I'm very happy with that. Thus, it wasn't too expensive, the total cost was maybe 15,000 euros and I had to pay 3,000 euros. (P12:52–57; male, age 50–54 years, implant-supported single crowns and implant-retained fixed partial dentures, pKV)  I have supplementary dental insurance. So I knew that I was relatively well insured and didn't worry about it. […] The dentist came straight away with the bill: “This and that will be covered by the health insurance company.” Since I know what my additional dental insurance covers, it wasn't a big challenge. (P05:57; female, age 55–59 years, implant-supported single crown GKV + suppl. insurance)  I had a supplementary dental insurance very early on because I knew, my molars were crowned and I was afraid that something could happen when I’m getting older. […][it] also covers a certain amount for implants. […] The whole was affordable for me. It is of course expensive. […] If you need maybe five or six [implants], then things are different. (P04: 33; female, age 55–59 years, implant-supported single crown, GKV + suppl. insurance) |
| **Costly but worth the investment** | It cost a few thousand euros and if you're a pensioner, that's of course a lot of money you have to pay. [...] But it was still worth it. I would do it again at any time. (P15:45; female, age 65–69 years, implant-supported single crowns and implant-retained fixed partial dentures, GKV + suppl. insurance)  Almost 12,000 [euros] we had paid. We were lucky that we had a [capital] insurance that could be cancelled that time. [...] I had a good experience at the university years ago that you can make partial payments. [...] then you're also a bit calmer about it. [...] My wife knows this too, my children too, they said: “Oh, that’s quite a lot!” But the quality of life, honestly, I have to say, has improved. (P11:34–37; male, age 65–69 years, implant-retained fixed prosthesis, GKV)  Well, I'm by no means swimming in money. But that was a priority for me. [...] I had saved something for a nursing home. I don't have any children and live alone. So I have to be prepared for a situation like that coming up at some point, and I had to draw on these financial reserves. But it also made sense, it was basically something similar, it was something medical. And it was important to me so that I didn't think about it too much. (P09:36–39; female, age 80–84 years, implant-retained fixed partial dentures, pKV)  Of course, it's also a question of cost. It's quite [expensive], goes up to 9,000 or 10,000 [euros] depending on what they do. And you think, yes, that's a lot of money but actually it's the money worth it. (P07:39; male, , age 70–74 years, implant-supported single crowns, GKV)  The health insurance company only pays little. [...] but I didn't really care what it costs in the end. Of course, I think these private fees are expensive but in the end that didn't matter to me. Well, I wanted to be well cared for, I was well cared for and that was okay. (P02:57–60; male, age 70–74 years, implant-supported single crowns, pKV) |

*Note.* GKV = Gesetzliche Krankenversicherung (statutory health insurance); pKV = private Krankenversicherung (private health insurance).

*Subtheme: Treatment procedure and healing process*

**Supplementary Table S9:** Perceptions of the treatment procedures

| **(Sub)Code** | **Exemplary quotes** |
| --- | --- |
| **Less demanding than anticipated – went great** | A bone augmentation wasn’t needed. That's exactly what they found in this examination. A doctor came in and said: “That looks good.’” And then he put the implants in straight away. [...] I wasn't prepared for it at that moment. I had said to my wife: “I have to have a check-up, bone grafting yes or no.” And then I came home and she looked at me: “What happened?” I just mumbled and I said: “I have the implants in.” [...] But, as I said, it went great. (P11:31; male, age 65–69 years, implant-retained fixed prosthesis)  I needed six [implants], which were then connected to each other with bridges. […] These six were put in all at once, I said to myself: “It’s definitely unpleasant, but then I don’t need to get step by step.” […] That worked perfectly. (P09:3; female, age 80–84 years, implant-retained fixed partial dentures)  I think it was definitely a year, everything together now with the implant consultation, the extraction, that went relatively quickly, then you had to come in a few times to adjust the crowns and so on. [...] It took a while. [...] But, as I said, everything was great. [...] I was amazed at how little pain I actually had, how relatively painless the whole [thing] was. I imagined it would be worse, much more worse. (P15: 15–35, 61; female, age 65–69 years, implant-supported single crowns and implant-retained fixed partial dentures)  A remainder of the tooth had to be extracted first. […] It had to heal first and then Mr. A. [dentist at the dental clinic] installed this pen there. And then the crown was made. And then Ms. B. [dentist at dental clinic] installed it there. […] Everything was perfect. (P27:8; female, age 65–69 years, implant-supported single crowns)  First, the old crown was removed and it had to heal then. I got temporary devices that I could chew with, also when the implants were placed but the crowns weren't yet on. [...] The pain was limited. [...] Everything went perfect, so there were no complications. [...] I had it [a bone transplant], too. [...] It was also without any problems. (P21:12; male, age 75–79 years, implant-supported single crowns and implant-retained fixed partial dentures)  I was relatively full of trust and it turned out that it was completely fine. Of course, these [therapy steps] were always longer periods, this for a month, then another two months and another two months and so on. (P02:52–54; male, age 70–74 years, implant-supported single crowns)  There were no problems at all, I tolerated it all well, Professor B. [dentist at dental clinic] [...] explained to me that this bone had to be exposed cleanly so that no residual connective tissue was incorporated and apparently he succeeded. (P17:7; male, age 70–74 years, implant-supported single crown) |
| **Long, demanding process but well performed and coordinated** | First of all, I felt being in very good hands. […] First, the upper jaw and lower jaw were adjusted to each other a little so that I got a proper bite again. This took two or three months. And then he [dentist] worked out the position of the implant with Professor A. [dentist at the dental clinic]. […] All in all, it took me a good six months before the operation could take place. Yes, and after the operation itself, I was a bit deformed in my face for three weeks, but the operation was very unproblematic. So everything went very well. […] And the whole thing took another, well, three months until it was completely grown in and the gums and […] everything was back to normal. And then we moved on to the prosthetics. […] It worked brilliantly, it was well coordinated. (P25:7–13; female, age 65–69 years, implant-retained removable prosthesis)  In the dental clinic, everything is tip-top, you can't say anything about it, it works exemplarily, wonderful. […] [Appointments] are also kept, [...] with minimal waiting times. It's very well organized. (P27:51–57; female, age 65–69 years, implant-supported single crowns)  When I was being treated, they told me what was going to happen next and so on. And the first time I had this bone build up, Dr. A. [dental surgeon], said ‘Now we're doing this and now I'm doing that.’ And then I thought, now he's starting and he was already finished. I couldn't believe it myself, [...] I can't say anything that could have been better. […] All I know is that I was terribly afraid of this bone augmentation, really, and was completely amazed that he said, yes, he's finished. (P01:33–35; female, age 85–89 years, implant-supported single crowns)  Well, it was really excellent. So first of all the preparation was great. […]. Of course, it was still stressful for me because my lower jaw turned out to be too narrow during the [preliminary examinations]. […]. A bone augmentation was made, something was removed from the back part and then added to it. And so I had to go around with very long-term temporary dentures for quite a while, because then they have to grow in first. […] I was of course incredibly reassured when I was pushed into a real operating room […]. With the things [heart problems] I had at that time, that if something happens to me there, I can be saved just down the hall. […]. Well, all the conditions were so great, I felt completely safe and everything went smooth. (P23:4–9; male, age 70–74 years, implant-supported single crowns) |
| **Stressful procedure that had to be endured** | I had a serious operation in 2017 because of a squamous cell carcinoma. […] The entire left upper jaw [was] removed […] A year later, the doctors at A. [dental clinic] managed to transplant bone from my left arm into the hole in my upper jaw. […] The transplant had to heal first. [...] They suggested to screw a special frame into the bone, if everything went well, teeth could be attached to that frame. […] That happened in 2019. I had implants inserted in the still existing upper jaw on the right. […] Then everything had to heal. […]. Then the prosthesis was made. […] The whole thing lasted until February 2020 until I had a full set of teeth again and was finally able to eat properly again. (P19:7–9; female, age 90–94 years, implant-retained fixed prosthesis)  The tumor was removed, then a year later pelvic bone was removed and inserted [into the jaw]. Everything had to heal first and then the implants were drilled into the new bone, had to grow in and then the dentures came on top. […] That were three years until I had the dentures, of which one year was for the bone transplantation, then another year, one and a half year for the implant, and until the teeth were on it about a year. […] At that time I had no teeth there. […] It wasn't so nice. It was extremely difficult to chew and especially to drink. (P06:10–11, 60–67; male, age 50–54 years, implant-retained fixed prosthesis)  That [implant insertion] was very stressful. And I was in the hospital one night after the operation. [...] After the operation you couldn't eat or drink as well. That is bad. (P14:12; male, 65–69 years, implant-retained removable prosthesis)  Well, of course you underestimate it. […] There is a lot of pre-treatment, were you have to open your mouth a lot, especially in the back molar area, so that the dentist can work in there as well as possible. (P04:51; female, age 55–59 years, implant-supported single crown)  Then he [dental surgeon] had to do a major operation, which was necessary because the bone had already decreased, and had to be rebuild. [...] Well, what I had to endure: keeping my mouth open for hours and this fear that you have about whether everything will go well. And then the hammering to insert the implant [...]. Well, it was stressful, of course. (P18:9–13; male, age 80–84 years, implant-supported single crowns)  (It) was more like this: “I'm sitting on a chair somewhere and have to put myself in the hands of them.” That's always unpleasant situation. […] when I was covered sterile, my head was wrapped up, so it was really just that mouth there, I thought: “No, stop, stop, stop, that’s enough for me now!” It was an overwhelming moment. (P26:8; male, age 55–59 years, implant-supported single crowns) |

**Supplementary Table S10:** Perceptions of symptoms and healing process

| **(Sub-)Code** | **Exemplary quotes** |
| --- | --- |
| **Minor normal temporary symptoms** | The healing process itself that day was great. I had no restrictions. Of course, it was swollen and all that, but it went really well. (P03:7; female, age 55–59 years, implant-retained fixed partial dentures)  Well, I only had a bruise on my face. But you knew that before. […] After three days it was good again. (P27:8–19; female, age 65–69 years, implant-supported single crowns)  When implants are placed, you are handicapped at first and you have to let them grow in for six months. […] You look a bit deformed but that's something else, you don't necessarily have to go out among people, you can stay back for two or three weeks. (P25:41; female, age 65–69 years implant-retained removable prosthesis)  Well, [it was said] that there could be some pain, some bruising, that there could be a rejection. But none of that really happened in my case, so it was unspectacular, uneventful, quite normal. (P17:35; male, age 70–74 years, implant-supported single crown)  I was amazed at how little pain I actually had, how relatively painless the whole [thing] was. I imagined it would be worse, much more worse. […] I probably have pretty good healing. Well, it always heals relatively quickly and I'm completely amazed at how quickly it actually healed and, yes, hardly caused any pain. So, after the operation, it was a little swollen on both sides, but everything was under control, it wasn't blue, there was nothing at all. It was really well done. (P15:61, 87; female, age 65–69 years, implant-supported single crowns and implant-retained fixed partial dentures)  I had black eyes and so on, that's normal. […] But it wasn't that bad. […]. He [the dentist] gave me some medication, but I didn't take it often […]. Well, the doctors think that something can happen, pain and that, but I didn't have any pain afterwards. (P07:29–37; male, age 70–74 years, implant-supported single crowns)  Of course, the first few days, you probably know that from others, these biting difficulties that you have then. I didn't really have pain at all. I was very surprised by that. Well, I cooled [may face] and hung around here for two days. But I have to say, it is a top matter. […] There was really only a slight bruise on the cheek. But otherwise there was nothing, no. (P11:7–11; male, age 65–69 years, implant-retained fixed prosthesis) |
| **Manageable symptoms and restrictions** | That was mostly okay. […] I concentrated on the other side while chewing. That wasn't something that couldn't be mastered. (P26:8ff; male, age 55–59 years, implant-supported single crowns)  That [implant insertion] wasn't a problem. […] Of course I had some swelling on my face. But I always cooled it, followed the instructions exactly and then after a few days it was fine. (P22:14–19; female, age 75–79, implant-supported single crowns and implant-retained fixed partial dentures)  Of course, after the operation everything was swollen. That lasted a week, then the swelling slowly went down, the bruises were visible for a little longer. But I actually didn't have any pain. […] During that time you could eat noodles well, and you could eat rice well, anything that was a little softer. But after a week you could eat something a little more solid again. And that took maybe four weeks. So, I didn't find the restriction on eating to be that serious. […] The pain was very, very bearable. (P25:7–17; female, age 65–69 years, implant-retained removable prosthesis)  Of course, it was a weird feeling first because it was a little thick. But it wasn't like I was in pain or that I would have said “I’m walking on my gums” or anything like that. So, it was okay for me. (P16:6; male, age 45–49 years, implant-supported single crown)  Sure, it was a bit difficult at the beginning with this temporary denture with the food and so on, but it worked. […] If something was broken, you could have it reworked any time. It was all problem-free. (P15:23; female, 65–69 years, implant-supported single crowns and implant-retained fixed partial dentures)  I knew what was coming and that it would take a long time and that wasn't so bad. […] With these temporary dentures I avoided things that I normally like to eat, like a really crunchy bread roll, […] but it wasn't really a big thing. (P09:3–21; female, age 80–84 years, implant-retained fixed partial dentures)  Of course, it was still stressful for me because my lower jaw turned out to be too narrow. […] A bone augmentation was made, something was removed from the back part and then added to it. And so I had to go around with long-term temporary denture for quite a while, because they have to grow in first. […] We had planned to go on a long boat trip. We got a suitcase from the A. [dental clinic], a small bag (with a repair kid for the temporary denture). […] Everything went smoothly. (P23:4–9; male, age 70–74 years, implant-supported single crowns) |
| **Burdensome and difficult-to-manage symptoms** | That was very stressful. […] After the operation you couldn't eat or drink as well. That is bad. […] After the new teeth came in, I was able to chew slowly and eat first things like soup, […] little by little until you can chew properly. (P14:15; male, age 60–69 years, implant-retained removable prosthesis)  That [the reconstruction] were a lot of steps. […] I couldn't speak or eat for a year […] only soup and things like that. […] It was a long, painful healing process. But with tablets it was bearable and the pain could be controlled. […] Of course, [I wasn't] well, had depressions at times. […] Thanks God my husband cooked, looked after me very lovingly. […] And sometimes I ate baby food in the beginning, anything you can swallow without using your jaw. (P19: 7–9, 62–73; female, age 90–94 years, implant-retained fixed prosthesis)  I had a lot of difficulties with this bone graft afterwards, it was terrible. [...] I just had the whole time extreme facial pain on the side. It had somehow attacked the facial nerve, or whatever. […] I had been on sick leave for 14 days, so I just lay in bed and cried. My face was so swollen, I was actually with the normal doctor [family physician], who said, it's like after a broken jaw. […] I was lying in bed crying for 14 days. I honestly thought it wouldn't get any better anymore, it was terrible. (P10: 10.; 66–67; female, age 55–59 years, implant-supported single crown)  I had no teeth there. […] It wasn't so nice. It was extremely difficult to chew and especially to drink. […] A lot of nerves were also removed and so the lips couldn’t hold the glass. That's why I had to drink everything with a straw. (P06:60–67; male, age 50–54, implant-retained fixed prosthesis) |

**Exemplary quotes – Theme 3: Living with implant-supported dentures**

*Subtheme: Perceived outcomes*

**Supplementary Table S11:** General outcomes of the dental implant treatment

| **(Sub-)Code** | **Exemplary quotes** |
| --- | --- |
| **Implant best solution – regaining quality of life** | |
| Generally very satisfied | I'm actually very satisfied. So I have, I don't notice any problems. [...] Well, it's just that you have to take a little more care of it. (P16:15; male, age 45–49 years, implant-supported single crown)  Really, I only can say good things. From the treatment itself, of course, and about the result. (P24:74; female, age 80–84 years, implant-supported single crowns)  All I can say is that I'm totally happy with everything so far. (P18:39; male, age 80–84 years, implant-supported single crowns)  Now that I have it all behind me, I have no pain and, yes, I'm as happy with my situation as one can be. (P19:9; female, age 90–94 years, implant-retained fixed prosthesis))  Well, I'm very satisfied. Now I can chew and eat almost everything [….] I don't have any complaints at the moment. I am very happy with the implant, [...] also in terms of appearance. You can't complain about that. (P14:23–27; male, age 65–69 years, implant-retained removable prosthesis)  It's completely fine, I'm very satisfied with everything. (P13:32–33; male, age 80–84 years, implant-supported single crowns and implant-retained fixed partial denture)  So it's really, it's really perfect, the implant itself, I was actually very happy with it. (P08:48; male, age 65–69 years, implant-supported single crowns)  Then I have got five implants. And I was satisfied because it was no longer this removable thing. (P24:14; female, age 80–84 years, implant-supported single crowns)  It was in the upper (jaw), […], the second tooth, next to the main incisor on the right, where the bone had to be built up somehow. And that was an operation that lasted, I don't know, more than an hour. But he did it brilliantly, this man. And *smiling* I was completely thrilled that an implant fit in there, afterwards. (P02:52–54; male, age 70–74 years, implant-supported single crowns)  I don't believe it myself, well, I don't have any problems! And when I hear from the others that they are experiencing a theater. [...] I have no problems at all. Now I got another implant, [...] thanks God I don't have any problems either, [...] that's really wonderful, it's like a miracle. [...] Well, I can't say anything that could have been better. (P01:8–13, 34; female, age 85–89 years, implant-supported single crowns)  That's all good, the (implant) itself is completely without problems and still fits well, is solid. (P10:29; female, age 55–59 years, implant-supported single crown) |
| Having firm teeth again | With these dentures, the removable ones, it was always that they became wobbly, when I chewed it became loose. [...] I was in the dental clinic almost every month and had to have it reworked and relined. It was a bit tedious. Now since these things [implant supported dentures] have been in place for a year and a half, it’s a completely different quality of life for me. (P11:15; male, age 65–69 years, implant-retained fixed prosthesis)  It's a pleasant feeling when you know that your teeth are firmly in place and won't fall out with any movement. [...] Well, that was a good decision. [...] I'm very happy that it [implant-supported prosthesis] is well anchored, [...] I can only recommend it. (P25:15; female, age 65–69 years, implant-retained removable prosthesis)  I'm very satisfied and I think it's great. [...] my teeth are all much firmer with the implants than my others. (P02:24; male, age 70–74 years, implant-supported single crowns) |
| **Integration into the body image** | |
| Integral part of the body | It's such an integral part of the body that I don't perceive it as different [from my own teeth]. (P23:25; male, age 70–74 years, implant-supported single crowns)  For me it's like my own tooth. So if I don't think about it at all, I don't even think it being an implant. (P04:17; female, age 55–59 years, implant-supported single crown)  Well, I had read about it on the internet before. It was said that the implant is like a tooth, a real tooth. And that has been confirmed. The implant, at least the one that I still have, can hardly be distinguished from the normal, grown tooth, it is basically equivalent. (P08:14; male, age 65–69 years, implant-supported single crowns)  I don't even notice that there's a foreign body in my mouth. (P12:49; male, age 50–54 years, implant-supported single crowns and implant-retained fixed partial dentures)  I don't notice that they are implants. Every now and then there's a slight sensation, that's probably because the gums have receded a bit. But it’s no pain now. It reminds me every now and then, that there's an implant. (P21:35; male, age 75–79 years, implant-supported single crowns and implant-retained fixed partial dentures) |
| Foreign body | An implant, the one top right, when I brush my teeth there, it doesn't hurt, not at all, but when I brush my teeth there, then I notice the implant, or whatever around the tooth. [...] I sometimes feel very tense in my cervical spine. And sometimes I have the feeling that I notice my implants, not that it hurts, no, just that I feel them. [...] Well, it's not that they were placed badly or anything, not that. […] I think it's a little bit related to the tension in the body, the head, the cervical spine or whatever, I don't know, I guess. (P15:35–39; female, age 65–69 years, implant-supported single crowns and implant-retained fixed partial dentures)  The question is, why does this implant feels so cold and so hard? I have all my molars crowned and I have these crowns, they are also cold and hard and yet I don't have the feeling that they are anything foreign. These crowns have always somehow been part of me. Well, of course, there are nerves involved. [...] I just wish this tooth wasn't so cold and so hard. So these are my feelings about this tooth. [...] With your tongue, it just feels different from the other crowns, but maybe that's my head, I don't know. (P05:58–65; female, age 55–59 years, implant-supported single crown) |
| **Decision for or against an implant in the future** | |
| Would choose it again | If that happens again, I would choose it again because, as I said, from my point of view it is the simplest, safest, easiest to maintain and best. (P27:37; female, age 65–69 years, implant-supported single crowns)  Well, yes, probably I would choose an implant again. [...] If it's possible to use an implant instead of dentures which you have to take in and out, I would prefer an implant. (P21:49; male, age 75–79 years, implant-supported single crowns and implant-retained fixed partial dentures)  I would choose it again at any time because it is simply very, very comfortable to wear. So you feel safe because the prosthesis is very tight. You don't have a plate under the palate in the top of your upper jaw, so your sense of taste is not restricted. [...] I would do it again because I'm just very happy with it. (P25:41; female, age 65–69 years, implant-retained removable prosthesis)  It did well, has held up well until today, everything is fine, no problems and, yes, I can only recommend: “Do it that way!” (P18:19; male, age 80–84 years, implant-supported single crowns)  I would decide for it straight away, so if it worked in the same way as it has so far in surgery and in everyday [life], I would take an implant again straight away. (P17:37; male, age 70–74 years, implant-supported single crown)  Well, I'm so glad I had it done, even though it cost a few thousand euros and if you're retired that's obviously a lot of money, you ultimately have to pay. But it was still worth it. I would do it again. (P15:45; female, age 65–69 years, implant-supported single crowns and implant retained fixed partial dentures)  I had it done and since then I've actually been happy. On the right side I thought to have conventional dentures. But that's not the right thing. Well, the best thing is an implant. [...] An implant is the best thing there is. I also told my friends: “If you still want to enjoy life, you have to have an implant.” (P07:4, 53; female, age 70–74 years, implant-supported single crowns)  Well, I would definitely choose an implant again, hoping that everything goes that well again. (P04:31; female, age 55–59 years, implant-supported single crown)  I got along quite well and can only recommend it to everyone. (P03:5; female, age 55–59 years, implant-retained fixed partial dentures) |
| Preferable option but carries considerable risks | Well, the implants I have are fine. I got the last ones from Professor B. [dentist at the dental clinic]. And that went completely smoothly [...] I hope I don't have to do that again. Because of course it is a very lengthy process and at my age, it was very time-consuming, yes. [...] The last one was completely unproblematic. But of course at my age, I'm taking blood thinners because I had a heart attack, I would of course think about it again very carefully. [...] It just is a question of age. When you're young, implants are actually the best solution. That's just how it is. (P22:20–29, 41; female, age 75–79, implant-supported single crowns and implant-retained fixed partial dentures) |

**Supplementary Table S12:** Outcomes in terms of appearance

| **(Sub-)Code** | **Exemplary quotes** |
| --- | --- |
| **Appearance great** | |
| Delightful appearance | That [the implant] works wonderfully in terms of appearance, too. You can't complain about it. (P14:26–28; male, age 45–49 years, , implant-retained removable prosthesis)  [The appearance is] great, the teeth are, well, they seem to be smaller now. At the beginning my wife said they were too small. But that was because for years I always had dentures on top, which kept getting bigger because they were relined. (P11:16–17; male, age 65–69 years, implant-retained fixed prosthesis)  If you would see it, they [the implant supported crowns] are so great, I am absolutely delighted with my appearance. (P01:21; female, age 85–89 years, implant-supported single crowns) |
| Like natural teeth | They [the implants] are molars, […] you hardly see them, they're further back and I have to say, if I don't get reminded from time to time, when I have to go to the dental cleaning or something like that, then I don't even notice them. They became part of my body for many years now. (P23:17; male, age 70–74 years, implant-supported single crowns)  [The appearance of the implant is] completely unspectacular […] I don't notice that a foreign body has been installed in the upper jaw. So, yes, it is ultimately like your own tooth. (P17:12–15; male, age 70–74 years, implant-supported single crown)  I am absolutely satisfied. [...] When all the crowns were inserted, […] I was really in the mood to party. There was nothing removable anymore, all the crowns were there and it looked as if they were my own, which once grew on me. (P09:17; female, age 80–84 years, implant-retained fixed partial dentures)  Well, I had read about it on the internet before and it was said that the implant is like a tooth, a real tooth. And that has been confirmed. Well, the implant, at least the one that I still have, can hardly be distinguished from the normal, grown tooth, it is basically equivalent. (P08:13–18; male, age 65–69 years, implant-supported single crowns) |
| **Appearance improved** | |
| Visual improvement | Of course, I also wanted a visual improvement and all of these things actually came true. (P26:25; male, age 55–59 years, implant-supported single crowns) |
| Teeth/crowns adjusted | In terms of appearance, with the fixed denture, that's really great. […] That worked really well. […] Also with Ms. A [dentist at the dental clinic], who kept saying: “Oh, let's look at it again and the color gradient too.” […] Due to previous denture-treatment my canines were a little bit up and the rest was a little bit down. That looked a bit strange. And then I talked with her and asked: “Can’t you do it so that it’s a little bit more similar to it used be before?” And then she said to me: “Yes, do you have any pictures?” […] Then I gave [her] a picture portrait and then she said: “Yes, we'll manage it like that.” […] That's really how it was afterwards or it seems like it was before. So if you don't know it, you don't see that I have dentures in there. (P03:15; female, age 55–59 years, implant-retained fixed partial dentures) |
| Upper/lower jaw adjusted | Professor A. [senior dentist in the treating dental clinic] also accommodated my wishes, he pulled my teeth up a bit, yes, they were a bit back up there. And now I have a really good facial appearance, top and bottom fit together perfectly, so it doesn't collapse like you often have when you get a conventional prosthesis. So it has become a completely normal facial appearance. […] I'm very happy with it, yes. (P25:18–21; female, age 65–69 years, implant-retained removable prosthesis) |
| Like new teeth | I have no restrictions. When eating I don't notice any restrictions and the appearance is like new teeth, like when I was eighteen. (P12:24–25; male, age 50–54 years, implant-supported single crowns and implant-retained fixed partial dentures) |
| **Appearance imperfect if not impaired** | |
| Not perfect but hardly visible | From a visual point of view it was great, there was a tooth again, but the new tooth didn't fit at all, neither in terms of the color nor in terms of appearance with the others. […] Well, in principle it [should] be adapted to the shape of the other teeth. I have this bone recession along with gum recession, […] now, I have a tooth in there like someone who is 20 years old and it just doesn't fit in with the rest. [...] You could just have shaped it differently. […] The color doesn't match either. Well, but it is not visible that much. (P05:21–27; female, age 55–59 years, implant-supported single crown)  Well, it is in the back of the cheek, so you can't see it when you laugh or something like. […] When you have your mouth wide open you can see where this little screw is from the implant, […] at the beginning it was also closed so that you couldn't see it. But then it loosened up a bit and then she [dentist] said that if it wouldn't bother me, she wouldn’t close it again, just leave it like that. (P04:12; female, age 55–59 years, implant-supported single crown) |
| Appearance impaired | The implant is in the front, […] the gums are pretty far down there. […] I haven't smiled since then anymore because one tooth is completely pushed inwards, and downwards the other one, […] so, the two sides look completely unequal. […] It was really a traumatic experience. […] I'm not medically knowledgeable, it was said that the crown had to go down that far so that everything was covered or whatever, so that it is closed. But it just out of place. (P10:33–38; female, age 55–59 years, implant-supported single crown) |

**Supplementary Table S13:** Outcomes in terms of function

| **(Sub-)Code** | **Exemplary quotes** |
| --- | --- |
| **Like natural teeth** | I can eat everything, chew it wonderfully. They perform the same function as your own real teeth. (P27:23; female, age 65–69 years, implant-supported single crowns)  It is all fine. The [implant] itself is completely problem-free. It still fits well, its solid. (P10:29; female, age 55–59 years, implant-supported single crown)  I don't notice anything [when eating], […] that these aren't my own teeth. (P01:18–19; female, age 85–89 years, implant-supported single crowns)  It's not different from before, I can bite with it in the same way, and I don't have to say: “Oh, now I have to be careful or something.” I mean, I wouldn't do things now that I wouldn't have done before with my normal teeth. So it's exactly the same as before. (P03:27; female, age 55–59 years, implant-retained fixed partial dentures) |
| **Able to eat properly again** | Thanks to this telescopic prosthesis and the implants, I can eat everything, there is no problem. (P22:31; female, age 75–79, implant-supported single crowns and implant-retained fixed partial dentures)  Well, now I'm very happy.. I can chew and eat now almost everything. It is perfect. [...] Everything works fine, so I can even chew nuts, chew them properly and eat them properly. (P14:23–29: male, age 65–69 years, implant-retained removable prosthesis)  My expectation was actually that I would be able to bite powerfully again. And I can do it. (P12:40; male, age 50–54 years, implant-supported single crowns and implant-retained fixed partial dentures)  The hope was that I would be able to eat and chew things like I did before the operation. And somehow that [was] completely fulfilled. (P06:19–21; male, age 50–54 years, implant-retained fixed prosthesis) |
| **Normally but careful** **eating** | I can eat normally, even crusty bread. I can eat my apple, too. [...] But I don't do that biting into a whole apple and then taking a bite out of it. [...] I could do it as Professor A. [dentist at the dental clinic] told me. [...] I'm still a little scared. [...] That's because you're branded when your teeth broke off when eating just normally. (P25:22–25; female, age 65–69 years, implant-retained removable prosthesis)  Well, of course I didn't eat apples on the first day of the implant insertion and did no tire test to see if it would hold. [...]. I put a little more strain on this implant for a few days and it went very well, there were no problems at all. (P17:9–11; male, age 70–74 years, implant-supported single crown) |
| **Temporary restrictions** | Well, and I can eat everything again in very small bites and the left side, I have to be careful that nothing comes over there, because then I choke very quickly. But I've gotten used to it. It takes twice as long for me to empty my plate as my husband, for example. But well, I have the time. [...] You get used to it. It's clear that you can't spontaneously eat everything like you used to do. But the fact that it even works is great. (P19:11–17; 25–29; female, age 90–94 years, implant-retained fixed prosthesis)  What annoyed me a little, was that I often bit my cheek while chewing. [...] You chew differently and it had to settle in first. But I have to say, things have gotten better little by little. That took a while, but that's just the way it is. (P11:17–19; female, age 65–69 years, implant-retained fixed prosthesis)  For me it's like my own tooth. [...] Well, the only thing that caused me difficulties was that I initially had a lot of pain in my jaw joint when chewing. I couldn't (eat) any solid foods, i.e. bite into an apple or a carrot, that didn't work at all, or chew a schnitzel. As soon as I tried to bite hard, it hurt a lot. But that was due to the jaw joint, not the implant as such. (P04:17–21; female, age 55–59 years, implant-supported single crown) |

**Supplementary Table S14:** Handling and self-care

| **(Sub)Code** | **Exemplary quotes** |
| --- | --- |
| Good oral hygiene as usual | Just normal dental hygiene. (P17:23; male, age 70–74, implant-supported single crown)  The handling is just normal. For seven or eight years I use interdental brushes. [...] I actually do that every day. [...] For a bridge that I still have, I work with dental floss, otherwise only with interdental brushes. I've had an electric toothbrush for probably ten years, one that's powered by a sensor. (P26:37–3; male, age 55–59 years, implant-supported single crowns)  This [handling and daily care] is no problem. [...] I use dental floss and everything, I've done that already before anyway. (P10:44–47; female, age 55–59 years, implant-supported single crown)  [For the daily hygiene], I have to pull out the replacement teeth first to clean them. You have to do this every day [...] in the morning and in the evening. You should also take the implant [-supported prosthesis] out twice a day and then clean it. [...] That’s not difficult, it's very simple. Just take it out and then clean it with the brush and then put it back in. (P14:31–35; male, 65–69 years, implant-retained removable prosthesis)  I have always taken good care of my teeth, but of course I take special care of the implant, I have these interdental tooth brushes and dental floss. (P08:20; male, age 65–69 years, implant-supported single crowns)  I have an electric toothbrush, but I only use it at the bottom of the teeth that I still have. Above I don't dare do that with the implants, I don't know why I use a normal toothbrush there, [...] and you always have to [clean them] with the dental floss. [...] I also use these little [interdental] toothbrushes there. [...] I brush with it in between, I always do it regularly and the lady who does my professional tooth cleaning, can always see that I'm doing it well. The other lady who did the check-up also said: “Well, everything was always very well cleaned.” (P15:50–53; female, age 65–69 years, implant-supported single crowns and implant-retained fixed partial dentures)  Even with all the cleaning, it's super easy. […] when I come to the dental check they tell me: “Oh well, it's really fine.” […] I have (inter-)dental brushes, they're a little bigger, […] you don't necessarily get them in the drugstore, but in the pharmacy, but that's not a problem. You can then clean the pillars and the gaps properly. Otherwise I just work with this floss. […] It's really easy to clean. (P03:11, 22–25; female, age 55–59 years, implant-retained fixed partial dentures)  It's like usual, after every meal and so on I have to clean my teeth and for the implant I have these [interdental] brushes, different brushes and that works. (P07:42; male, age 70–74 years, implant-supported single crowns)  Well, I actually look at the implant like my own teeth, that's how it feels to me. This means that I also carry out very thorough dental hygiene using an electric toothbrush and oral irrigator. (P04:13–15; female, age 55–59 years, implant-supported single crown)  I have done this before. [...] the process was always: I brushed the teeth, cleaned the gaps with the interdental toothbrush and finally used the oral irrigator. [...] In the dental clinic, where I go for prophylaxis now, I was told that I should continue like this. (P24:37–38; female, age 80–84 years, implant-supported single crowns) |
| Oral hygiene as recommended | They gave me different things, like this dental floss and show me again how to use it every time. [...] Of course we learned a lot that we didn't know before. [...] And of course that's more time-consuming, but in no way a burden, not disturbing, you just need a little more time. (P23:17–21; male, age 70–74 years, implant-supported single crowns)  Of course, I have interdental brushes, I have dental floss, I have an extra brush for brushing again. I have a special brush for the telescopic prosthesis. So I do everything that is discussed with me in the dental clinic. (P22:55; female, age 75–79, implant-supported single crowns and implant-retained fixed partial dentures) |
| Special oral hygiene needed but no effort | The implants need to be cared for, of course. So I just take out the two dentures, brush my teeth as normal and of course, with an interdental brush, with a small one, so that the spaces are cleaned. [...] I was shown how to do it and I have the appropriate sizes for the gaps. And it works, they were always very happy with the dental care, so it seems like I'm doing it right. [...] That's not a lot of effort. I take a soft toothbrush, brush the implants as normal, and at the end I go between them with this interdental brush and clean the gaps, three times a day if ever possible. ... Once you have experienced such a catastrophe, you naturally become particularly careful and try to preserve everything for as long as possible. And as I said, it's really not that much effort. It's a well-rehearsed routine. Thus the dental hygiene is actually quite normal. (P25:31–35; female, age 65–69 years, implant-retained removable prosthesis)  Dental hygiene with tooth brush and floss, just normal, it is not different from having normal teeth. [...I] didn't use dental floss so intensively before. I don't do it every day, but several times a week. I didn't do that with dental floss before, only with a toothbrush. (P21:41–43; male, age 75–79 years, implant-supported single crowns and implant-retained fixed partial dentures)  I've now the habit of putting it in an ultrasonic bath in the evening to clean it, which is better than using a brush or something like that. And the implants themselves above, so cleaning them with dental floss is enough. I have to tell you to be honest, I have problems with that and now I actually do it with a brush for dentures, which is a bit thicker, but I then scrub the top and rinse properly. And I've never had a problem with it. (P11:21; male, age 65–69 years, implant-retained fixed prosthesis)  You have to take a little more care, especially at the root of the teeth and so on. [...] That's just the nature of the implant. [...] That sounds a bit stupid, but the implant was expensive and it's supposed to last a long time. So a bit like: Do it right and watch and so on. (P16:14–17; male, age 45–49 years, implant-supported single crown)  You have to take care of it. Always clean up after eating and so on. But so far it has worked well. [...]  I mean, every dentures does a little bit of work. I have to clean my implant after every meal, and it would be even worse with removable dentures. (P07:4; 20 male, age 70–74 years, implant-supported single crowns) |
| Oral hygiene worthwhile yet requires significant effort | I have to take it [implant-supported prosthesis] out after every meal. Due to the transplant it isn’t completely sealed, there's still a tiny gap something always slips in there behind the dentures. [...] and I clean it thoroughly and in the evening anyway. [...] Well, I have time, I'm retired. I got used to it. It actually always takes 20 minutes. (P19:18–20; female, age 90–94, implant-retained fixed prosthesis))  The toothpick is my constant companion, depending on what you eat. Cleaning my teeth in the evening is a ritual. It's a real act, it takes a long time, first brushing and rinsing, then flossing all over. And first remove the worst of it with a toothpick. And then brush and then rinse again with mouthwash and then the splint comes in at the end, the grinder splint. [...] Dental care was secondary for me, but since I had this dental operation and implants and so on, it has become a real ritual for me, which I take seriously. [...] First it's expensive and then of course you have to take care of your teeth, [...] your health also dependents on the teeth. (P18:51–57; male, age 80–84 years, implant-supported single crowns)  It requires more cleaning effort than with normal teeth. [...] Well, normal tooth brushing and then because of this disease I probably have to clean the gaps between the bridge and the gums, have to clean them very thoroughly with my interdental brushes. [...] It [the disk-implant) requires a lot of effort, […] because it is removable. […] But the advantage is that the gum is not attached to the tooth and if something gets in there and becomes infected, it won't immediately affect the bone, even though the implant would otherwise be screwed into the bone over a large area. (P06:23–25, 53; male, age 50–54 years, implant-retained fixed prosthesis)  Well, I do whatever I can to keep the teeth in order. […] With this electric toothbrush of course, I try to brush as best as I can. Then I use these little interdental brushes. And I have also a tongue cleaner and then I take Listerine as well, I put it on these [interdental] brushes before I put it in the gaps between my teeth. […] Well, I really try to do as much as I can myself. (P01:22–29; female, age 85–89 years, implant-supported single crowns) |

*Subtheme: Care requirements and complications*

**Supplementary Table S15**: Professional dental care

| **(Sub-)Code** | **Exemplary quotes** |
| --- | --- |
| **Regular dental control and professional tooth cleaning as usual** | |
| Regular prophylaxis and dental control as usual | I go to dental prophylaxis every six months and then everything is checked. […] I’ve done it already before. (P27:29–31; female, age 65–69 years, implant-supported single crowns)  I've always gone to the dentist every six months. […] Professional tooth cleaning is carried out as normal. Currently, I have no particular challenges with this implant. (P05:43–45; female, age 55–59 years, implant-supported single crown)  I go for tooth cleaning every six months and Professor A. [dentist at the dental clinic] usually looks to see if there are any problems. […] It's usually a combined appointment for tooth cleaning and check-up, that's one appointment. (P21:44; male, age 75–79 years, implant-supported single crowns and implant-retained fixed partial dentures)  I go there for prophylaxis once every six months and Doctor A. [dentist at the dental clinic] […] checks everything regularly to see if there are any symptoms of caries or something like that. […] I have exposed tooth necks and he looks if everything is still okay. […] In the dental clinic, they've been doing this [professional tooth cleaning] already for years, and I'm very happy with it. (P17:25–33; male, age 70–74 years, implant-supported single crown)  For prophylaxis I go twice a year and then once a year there is also a dental check-up. If I have problems with other teeth or something, I always go there. […] I'm still at the A. [university dental clinic], except for prophylaxis I'm somewhere else because of the supplementary dental insurance I have to go to a certain practice then. (P10:50–55; female, age 55–59 years, implant-supported single crown)  And of course I do this prophylaxis twice a year at A. [dental clinic], yes, and it works. Hopefully it stays like this. (P07:43; male, age 70–74 years, implant-supported single crowns)  I do this (dental check-up) with my dentist. […] But he does it so quickly and I'm always a bit skeptical. So I just find myself in better hands at the A. [dental clinic]. [...] There [in the dental clinic] I go for periodontal treatment. (P02:38–44; male, age 70–74 years, implant-supported single crowns) |
| Close dental control and prophylaxis to prevent or treat periodontal disease | Well, I also go every three or four months to a professional tooth cleaning to A. [dental clinic]. I really do what I can do to keep my teeth in order. […] I try to go to the tooth cleaning every quarter [of a year]. […] If they find something is wrong during the teeth cleaning […], then B. [dentist] comes and takes a look. He checks my teeth at least twice a year. (P01:23–29; female, age 85–89 years, implant-supported single crowns)  I have been treated for periodontal disease since I was 13 years old. […] I go for dental care every three month, and for example, tooth cleaning. (P22:47.; female, age 75–79, implant-supported single crowns and implant-retained fixed partial dentures)  This [peri-implantitis] is, of course, a risk. But I go for prophylaxis three to four times a year and get everything checked. I don't have any problems with that. […] I've done that [prophylaxis] for years already. (P13:33–41; male, age 80–84 years, implant-supported single crowns and implant-retained fixed partial denture)  I do both [dental control and tooth cleaning for periodontal disease prevention] all three or four months. (P12:33–39; male, age 50–54 years, implant-supported single crowns and implant-retained fixed partial dentures)  Well, I go for regular treatment to the A. [university clinic] already since 2013. […] Every four months I go there for tooth cleaning. And otherwise, follow-up care is always carried out. Of course, other problems arose in the meantime, apart from the one with the implant. (P08:33–44; male, age 65–69 years, implant-supported single crowns)  I do my prophylaxis twice a year at A. [dental clinic]. […] I still get a deep cleaning on average every two to three years, and of course this was also the case before my implants. (P26:39; male, age 55–59 years, implant-supported single crowns) |
| Constant dental treatment required | I am under constant treatment. Of course, I always have my teeth cleaned, at least every six months. But since I go to the dental clinic quite often, my teeth are constantly under control. And if there is something, then I immediately say there is something, it tweaks there and it will be dealt with accordingly. (P18:77; male, age 80–84 years, implant-supported single crowns)  The direct aftercare itself is done. Since recently I was for dental further treatment in the student course and Doctor C. [dentist at the dental clinic] always checked the [implant-supported] crown, too. The last time in February and said: “Everything is fine.” […] I'm happy with that at the moment. […] Thus, I was spending a lot of time at A. [dental clinic] anyway. I don't yet know how things will continue to work. […] In the treatment in the student course every time someone kept looking at my dental state. (P16:38–41; male, age 45–49 years, implant-supported single crown) |
| **Special implant-related dental control and guidance** | |
|  | I go to dental prophylaxis three times a year. […] that's new now, whenever I go for prophylaxis, I should also come to Professor C. [dentist at the dental clinic], or to Ms. D. [dentist at dental clinic]. (P24:38–40; female, age 80–84 years, implant-supported single crowns)  I always go for a dental cleaning and monitoring on a regular basis [...] and they gave me different things. There you have this dental floss, […] they also show me every time [how to use it]. And that's the only thing, I have to clean differently now. (P23:17; male, age 70–74 years, implant-supported single crowns)  Especially the situation with Doctor A. [dentist at the dental clinic] is very good. […] We always make the appointments so that he can come […] either before or after the prophylactic treatment. […] The implants or other things that have been made are always checked by those who have a professional eye on them. (P26:55; male, age 55–59 years, implant-supported single crowns)  Professor A. [dentist at the dental clinic] always looked at it [dental implant] regularly. […] He also constantly takes photos […] and, well, it's constantly documented. […] So I really feel like I'm in very good hands in the dental clinic. (P25:64; female, age 65–69 years, implant-retained removable prosthesis)  Now after four years an X-ray check was taken and everything looked fine. (P15:59; female, age 65–69 years, implant-supported single crowns and implant-retained fixed partial dentures)  Usually a year after [the implant-insertion] you have to go to the hospital again for a check-up, yes. (P14:31; male, age 65–69 years, implant-retained removable prosthesis)  I still go to Doctor A. [oral surgeon at the dental clinic] who placed the implants once a year. First it was every six months, now it's once a year and in the prosthetics every six months. And also to the special tooth cleaning every six months. It is very important that no bacteria get stuck in the mouth. Yes, and the special check-up every six months anyway. Well yes, I'm often at the A. [dental clinic]. (P19:23; female, age 90–94 years, implant-retained fixed prosthesis))  Normally, I usually go for follow-up care every six months. That has to do with the implants. But because of the peri-implantitis, I'm now on a smaller schedule, so I go there more often. (P06:30; male, age 50–54 years¸ implant-retained fixed prosthesis)  So at first there were very close dental check-ups, she [dentist at the dental clinic] looked at how the gums are, how I am getting along, then she also checked once, if everything is okay with the bite. And now I go there every six months just like I do for normal preventive care, unless I have something. (P04:22–23; female, age 55–59 years, implant-supported single crown) |
| **Neglected dental control and prophylaxis** | |
|  | I was a bit sloppy with that [dental control]. […] I should have gone there a little more often. Because caries developed again I made an appointment with the dentist who had done the implants. Of course, he looked at everything and one of the crowns wobbled a bit. I didn't take it particularly seriously at that moment and the dentist then told me: “If there's anything wrong with the implants, come in immediately! You can’t wait and say, ‘It’s just a little bit’.” So the aftercare is completely okay. (P09:24–27; age 80–84 years, implant-retained fixed partial dentures)  Well, I have to admit that last year I really omitted it [dental check-up], [...] otherwise I always go once a year. And as soon as I have problems, I tell him [the dentist] that, too. We have now also decided that we need to reline the lower jaw. And now he said that these little white holding parts all need to be replaced now. (P11:25; male, age 65–69 years, implant-retained fixed prosthesis) |

**Supplementary Table S16:** Complications and after care

| **(Sub)Code** | **Exemplary quotes** |
| --- | --- |
| **Complications** | |
| **No significant complications** | There were no complications. (P07:29; male, age 70–74 years, implant-supported single crowns)  Everything is actually fine, […] there have never been any major complications. (P26:39; male, age age 55–59 years, implant-supported single crowns)  I haven't had any complications so far and I'm very satisfied. And I don't drink or smoke. Well, I actually live a healthy life and I don't know if it has anything to do with that. Well, I can't, I don't even notice that there's a foreign body in my mouth. (P12:49; male, age 50–54 years, implant-supported single crowns and implant-retained fixed partial dentures) |
| **Implant/denture failure or loss** | |
| Denture/crown does not fit | I couldn't use it [implant-supported crown] for a very, very long time because I had no way of chewing with my teeth on the side. They had no contact at all anymore because at the other side this crown was too big. I have bad tooth structure anyway. This means that if I eat something harder, something will break down very quickly. I have large gaps between my teeth and a lot of food stays in them. So I have the habit of eating soft and mushy foods, anyway. […] About a year ago, a dentist finally recognized it and then finally reacted. Several [dentists] recognized it, but he was the only one who reacted. He grounded off three of my crowns so that I can now chew on that side again. (P05:35–41; female, age 55–59 years, implant-supported single crown) |
| Crown broke off | Well, there were problems afterwards when everything was finished because the laboratory had somehow made a mistake. The neck of this crown broke off after three months. And that was a bit annoying because the whole thing had to be made again. (P27:10; female, age 65–69 years, implant-supported single crowns)  One [implant-supported crown] broke after the warranty period. And that's the crux of the whole story at the moment. […] I don't know what happens with the broken one, I've an appointment in April for trying to get the screw out somehow. (P02:2; male, age 70–74 years, implant-supported single crowns) |
| Implant loss | The seven fell out six months later, I just had it in my mouth, painlessly. […] The four fell out again due to periodontitis about three years ago. I now only have two [implants] out of four left. […] The seven, well, that's right at the back, you can't see it, it didn't have to be […] it was an optional indication to implant the seven. But the four, I have a gap there. […] For restauration you would also have to remove the five implant because the four gap is very narrow and then put an implant on four and five. Due to my age and no lack of functionality, I don't want to have anything done to it. And that little gap is visible only when you laugh broadly, is something I can live with. (P13:29–31; male, age 80–84 years, implant-supported single crowns and implant-retained fixed partial denture)  At one molar periodontitis developed again and it [the implant] became loose and then it had to come out. […] I'm now thinking about what I'm going to do. It was suggested that another implant could be placed, but I see it like this: once it fell out, once it loosened, hmm, I'm thinking about that at the moment. […] The implant in the field of vision, which is much more important, is wonderful. […] The other doesn't actually bother me, it's out of sight. […] I can handle it. […] It's not that it wasn't done well, it's more about the fact that it didn't work once. […] Thus, I don't know whether I want to do that again. There are also considerable costs. […] It was already said from the beginning that these implants can also be affected by periodontitis again, that implants can become loose and that unfortunately turned out to be true for me with one tooth. (P08:20–44; male, age 65–69 years, implant-supported single crowns)  After two years the (fastening elements of the implant-supported removable) prosthesis became slightly loose. It had to be corrected properly. (P14:70; male, age 65–69 year, implant-retained removable prosthesis) |
| Denture damaged during dental intervention | I had another operation in 2019 and they cracked my dentures a bit, then I was in the A. [dental clinic] in the morning. Yes, it was taken out and then brought to C [location of the dental laboratory], they just took a look at it and did a little bit of refining and reworking and put it back in the afternoon. So that worked really well, too. (P03:21; female, age 55–59 years, implant-retained fixed partial dentures) |
| **Prolonged symptoms and after care** | |
| Post-treatment jaw joint problems and therapy | The only thing that happened after that was because of these many, lengthy treatments in the molar area. I have got problems with my jaw joint. It turned out that the disc was displaced. Well, I've had physical therapy, manual therapy with ultrasound, for over a year now. And, well, there's progress now, of course very, very slowly. But there is progress, so that I can push my lower jaw forward slightly again, […] and the pain has subsided. And I also went for an MRI, which the doctor from A. [dental clinic] had arranged. […] It was discovered that I have osteoarthritis in my jaw joint. […] My dentist already prescribed that [physical therapy]. One gets crazy if it cracks every time you open your jaw. She [general dentist] looked at it, checked again whether the bite was correct and all that and then said I had to do manual therapy because I said that I had these limitations with my jaw. Of course, she was very, very embarrassed, and she got very involved and said that I should do [some] manual therapy and I then said, I would really like led it check at the A. [dental clinic], that was also her suggestion. […] She supported me a lot with everything. (P04:3. 42–46; female, age 55–59 years, implant-supported single crown)  And it [jaw joint] has now somehow maneuvered itself into a position where everything is at rest. And if I have to keep my mouth open for a long time at the dentist, like when I get implants, then, of course, I have problems because it cracks again and it hurts. But then I always say that I have to close my mouth every now and then, […] then it's fine again. […] In the beginning I had real treatment for it. But then I let that go because, in my opinion, it didn't really do anything for me. Everything is fine now. Knock on wood! (P18:87–89; male, age 80–84 years, implant-supported single crowns) |
| Persistent pain –left alone | I had extreme facial pain on one side then all the time. The facial nerve or whatever somehow had been attacked… I was pretty much left alone with it. According to the motto: "No, everything is pathologically okay, nothing can be seen, everything fits well and everything is perfect." Well, it did fit, but that was there where the piece was taken out […] That was really terrible, I was pretty exhausted for five years, and I was unable to work for a while because it was that bad. […] Well, I took pills [...]. Actually I felt so bad that I wasn't able to take care of it myself at all. [...] I have often been to the doctor at A. [dental clinic] also to these implantologists, there were different areas in the dentistry. Both sides then said that everything was fine and there was nothing they could do about it and everything would be okay. Well, and that was terrible. [...] For further treatment I actually went to the family doctor and the neurologist. (P10:13–20; female, age 55–59 years, implant-supported single crown) |
